# *In cellulo* Evaluation of Phototransformation Quantum Yields in Fluorescent Proteins Used As Markers for Single-Molecule Localization Microscopy

**DOI:** 10.1371/journal.pone.0098362

**Published:** 2014-06-10

**Authors:** Sergiy Avilov, Romain Berardozzi, Mudalige S. Gunewardene, Virgile Adam, Samuel T. Hess, Dominique Bourgeois

**Affiliations:** 1 Université Grenoble Alpes, Institut de Biologie Structurale (IBS), Grenoble, France; 2 CNRS, IBS, Grenoble, France; 3 CEA, DSV, IBS, Grenoble, France; 4 Department of Physics and Astronomy, University of Maine, Orono, Maine, United States of America; The Beatson Institute for Cancer Research, United Kingdom

## Abstract

Single-molecule localization microscopy of biological samples requires a precise knowledge of the employed fluorescent labels. Photoactivation, photoblinking and photobleaching of phototransformable fluorescent proteins influence the data acquisition and data processing strategies to be used in (Fluorescence) Photoactivation Localization Microscopy ((F)-PALM), notably for reliable molecular counting. As these parameters might depend on the local environment, they should be measured *in cellulo* in biologically relevant experimental conditions. Here, we measured phototransformation quantum yields for Dendra2 fused to actin in fixed mammalian cells in typical (F)-PALM experiments. To this aim, we developed a data processing strategy based on the clustering optimization procedure proposed by Lee et al (PNAS 109, 17436–17441, 2012). Using simulations, we estimated the range of experimental parameters (molecular density, molecular orientation, background level, laser power, frametime) adequate for an accurate determination of the phototransformation yields. Under illumination at 561 nm in PBS buffer at pH 7.4, the photobleaching yield of Dendra2 fused to actin was measured to be (2.5±0.4)×10^−5^, whereas the blinking-off yield and thermally-activated blinking-on rate were measured to be (2.3±0.2)×10^−5^ and 11.7±0.5 s^−1^, respectively. These phototransformation yields differed from those measured in poly-vinyl alcohol (PVA) and were strongly affected by addition of the antifading agent 1,4-diazabicyclo[2.2.2]octane (DABCO). In the presence of DABCO, the photobleaching yield was reduced 2-fold, the blinking-off yield was decreased more than 3-fold, and the blinking-on rate was increased 2-fold. Therefore, DABCO largely improved Dendra2 photostability in fixed mammalian cells. These findings are consistent with redox-based bleaching and blinking mechanisms under (F)-PALM experimental conditions. Finally, the green-to-red photoconversion quantum yield of Dendra2 was estimated to be (1.4±0.6)×10^−5^ *in cellulo* under 405 nm illumination.

## Introduction

Single-molecule localization fluorescence microscopy [1], [2], [3], [4] has become a central tool to investigate cells with unprecedented spatial resolution. In (Fluorescence) Photoactivation Localization Microscopy (F)-PALM (hereafter referred to as PALM), genetically encoded phototransformable fluorescent proteins (PTFPs) are used to sequentially localize individual emitters with sub-diffraction accuracy, so as to reconstruct a super-resolved image of pointillistic character. During data-acquisition, the density of emitting fluorescent molecules depends on a number of photophysical processes, including photoactivation, reversible blinking and irreversible bleaching. A thorough characterization of these processes is particularly crucial in quantitative studies aiming at counting molecules, as recently shown for proteins forming multimeric cell machineries [5], [6], found in organelles of sub-diffraction size [7] or arranged in clusters smaller than the diffraction limit [8], [9]. (For a review see [10]).

In this work, we establish the feasibility of measuring phototransformation quantum yields of PTFPs directly in cells using standard PALM microscopy. A few previous studies have measured photobleaching yields of non-phototransformable fluorescent proteins at the single-molecule level, typically *in vitro* to ensure a sufficiently sparse molecular density [11], [12], [13], [14], [15]. A wide range of values (10^−5^–10^−6^) were obtained, sometimes for the same protein using different protocols [12], [13]. It was proposed that the experimental conditions and notably the employed single-molecule immobilization technique could alter the photophysical properties of the FP under study. In the case of green-to-red photoconvertible fluorescent proteins (PCFPs), an important PTFP's subclass of common use in PALM microscopy, an estimate of the photobleaching quantum yield of mEosFP was obtained (Φ = 3×10^−5^) using BSA surface-immobilization [16], and it was suggested in the case of mEos2 and pcDronpa2 [17] that bulk measurements of photobleaching yields may not be valid in single-molecule microscopy conditions. The photoconversion quantum-yield of PCFPs was measured in the case of KikGR [18] and its monomeric variant mKikGR [19] in solution or in gels, giving values of the order of ∼5×10^−3^. Other careful studies on e.g. Kaede [20] or EosFP [16] did not provide explicit values for photoconversion quantum yields. Recently, estimates of photoconversion efficiencies, another important parameter accounting for the total fraction of fluorescent molecules able to undergo a transition to the active (red) state, were obtained *in cellulo* for various PCFPs, but again no quantum yield was provided [21].

Since the seminal work by Dickson *et al* [22], the blinking behavior of a number of FPs was investigated by fluorescence correlation spectroscopy [12], [23], [24], [25], concentrating on fast (∼kHz) rather than slow blinking regimes (∼1–10 Hz). The occurrence of long-lasting dark states has been noticed [26], [27], [28], but no quantum yields for the corresponding *on-off* transitions have been reported. Long-lasting dark states of PTFPs deserve special attention because they are detrimental to PALM microscopy and single-particle tracking experiments. In PALM, clustering and molecular overcounting artifacts induced by slow blinking of mEos2 or PA-GFP have been recognized in a number of seminal studies [27], [29], [30]. Indeed, transient dark states with lifetimes exceeding the typical frametime in PALM data acquisition (10–50 ms) cause single emitters to appear several times during a given data acquisition. To correct for these blinking-induced artifacts, several strategies have been proposed based on grouping fluorescence bursts that appear close in space and time. Annibale et al [31] used an approach in which the fluorescence traces from spatially close spots are subdivided whenever an off-time exceeding a given threshold *t_d_* is reached, resulting in a number of sub-traces *N(t_d_)*. The true number of molecules is then obtained by fitting the *N(t_d_)* curve with a semiempirical model accounting for blinking. A variant of this technique based on grouping spots with Kalman filtering was developed by Lando *et al* [32]. To accurately group spots in both the temporal and spatial dimensions, Coltharp *et al* [33] used another empirical strategy based on calculating a Jaccard index as a function of two thresholding variables, *t_Thres_* and *d_Thres_*. The optimum *t_Thres_* and *d_Thres_* are then applied to the entire data set, independent of the local molecular density. Recently, an elegant method was introduced by Lee *et al* [8] that proposes to subdivide molecular fluorescence traces based on a cutoff value *τ_c_* which is dependent on the FP photobleaching and photoblinking rates as well as the local density of molecules. The optimum cutoff value *τ_c,opt_* giving rise to a minimal counting error is obtained by an iterative procedure, the actual number of molecules in each evaluated spot cluster being the unknown. In this approach, bleaching and blinking rates of the PTFPs are obtained from *in vitro* experiments in which the molecular density can be kept sparse enough to prevent spatial overlap between molecules. None of the above techniques, however, was designed to extract the actual photoblinking quantum yields of the employed PTFPs *in cellulo*.

Overall, although the importance of evaluating PTFP's photophysics in biologically relevant experimental conditions is increasingly recognized [7], [32], [33], a method to quantitatively evaluate their phototransformation yields in densely labeled cells is still lacking. Such *in cellulo* evaluation would facilitate accurate molecular counting and have a fundamental interest for the mechanistic investigation of FP photophysics and the future design of improved variants.

On the structural standpoint, the photoconversion mechanism of green-to-red PCFPs such as Kaede [34], Dendra2 [35], [36], mEos2 [37], mKikGR [19], mClavGR2 [38], mMaple [39], pcDronpa2 [17] and their variants has been well characterized, involving a light-induced breakage of the protein backbone next to the chromophore which results in irreversible elongation of its electron conjugation through a β-elimination reaction [16], [34], [40], [41]. In contrast, the precise blinking and bleaching mechanisms of these FPs remain incompletely understood, despite recent structural studies of IrisFP, a variant of EosFP [42], [43], [44]. Overall, investigations of blinking in FPs has been lagging behind those of organic dyes typically used in (direct) STochastic Optical Reconstruction Microscopy ((d)STORM) [45], [46], [47]. A large body of work has demonstrated that organic dye photophysics is strongly dependent on environmental parameters such as pH, presence of molecular oxygen or redox partners, attachment to protective agents [45], [47], [48] and even the nature of the molecule labelled by the dye [49]. Although the 4-(*p*-hydroxybenzylidene)-5-imidazolinone (*p*-HBI) chromophore in fluorescent proteins is to some extent protected from the external medium by the β-barrel matrix, recent evidence has been provided that such environmental dependence also applies to FPs. Redox agents [50], [51], [52], pH [23], [25], temperature [53], physicochemical parameters such as viscosity [54] and even external electric or acoustic fields [55] indeed have been shown to greatly modulate the photoconversion, photoblinking, photoswitching or photobleaching behavior of a number of FPs. Likewise, fixation conditions or type of fusion construct employed in biological cells could play a stringent role [56].

Performing *in cellulo* single-molecule measurements of PTFP's photophysics is not straightforward. Such measurements are typically hindered by unfavorable signal-to-noise ratio of the detected fluorescence, and by the potentially high density of labeled molecules. In PALM, due to residual activation by the readout laser beam, a sufficiently sparse distribution of activated molecules in each frame is not guaranteed. To extract phototransformation yields, the photoexcitation rates of individual molecules need to be assessed, which requires the precise knowledge of not only their extinction coefficients but also of the local laser power-density and polarization (which in turn complicates the use of a TIRF geometry). Fluorescent molecules might adopt a variety of orientations, and the strong dependences on dipole orientation of both photon absorption and fluorescence response need to be taken into account. Finally, the inhomogeneity of the chemical nanoenvironment in the cell may result in multiple photophysical behaviors coexisting within the sample.

Here, we propose a method to tackle these difficulties. To estimate the phototransformation quantum yields of green-to-red PCFPs *in cellulo*, we devised a strategy based on an extension of the *τ_c_* optimization approach of Lee et al [8]. The performance of our approach was evaluated by performing extensive simulations, so as to establish a range of experimental conditions for which the method is expected to work reliably. The phototransformation yields of Dendra2 fused to actin were then measured in fixed mammalian cells, and compared to those of Dendra2 embedded in a poly-vinyl alcohol (PVA) matrix, in the presence or absence of the antifading agent 1,4-diazabicyclo[2.2.2]octane (DABCO).

## Methodology

### Generation of simulated data

To generate simulated data, comprehensive PALM simulation software was written in Matlab (Figure S1, Scripts S1), with additional features as compared to similar software developed to date (see *e.g.* [31], [33], [57]). A chosen number of dye molecules are randomly placed on a defined image pattern (typically a segmented nanoscopy image), so as to control dye molecular density. The photophysical properties of the fluorescent markers (mostly phototransformable fluorescent proteins) are defined in a database containing parameters such as excitation and fluorescence emission spectra, fluorescence quantum yield, phototransformation action spectra as well as photoconversion, on-off photoswitching, photoblinking and photobleaching quantum yields. Properties of the PTFP's chromophores in their anionic and neutral states are distinguished and simulations at various pHs can be performed. The orientation of the chromophore dipoles can be selected as “tumbling” (emitters are freely rotating, representative of e.g. a live cell), “fixed” (emitters assume a random orientation, but constant over time, representative of e.g. a fixed cell), or “uniform” (emitters adopt the same dipole orientation, constant over time, representative of e.g. oriented molecules in a fixed or live cell).

The experimental setup is simulated by a number of parameters describing laser illumination, microscope optical properties and detector characteristics. Four laser beams with Gaussian spatial profiles within the focal plane can be defined (i.e. for activation, switching, back-switching and readout), with chosen wavelengths, powers, polarization (circular or linear) and FWHM (full width at half maximum). Complex laser sequence patterns can be generated during data collection, such as for example pre-bleaching steps with the readout laser, and ramping-up or a Fermi profile [8] for the activation laser. The microscope is described by an objective of given numerical aperture *NA*, which is assumed to give rise to a Gaussian point spread function (PSF) with standard full width at half maximum (*FWHM = 1.22λ/NA* where *λ* is the emission wavelength). The photon collection efficiency takes into account the orientation of the emitting dipoles based on Fourkas *et al* [58]. Transmission efficiencies of inserted emission filters, as well as overall transmission efficiency of the optical setup are also taken into account. The EMCCD detector is described by the effective pixel size, dark current noise, readout noise and gain (counts/detected photon).

PALM data sets with a defined frametime and number of frames can then be generated. For each single-molecule emission, the spatial (throughout the microscope PSF) and spectral (throughout the emission spectrum) distributions of emitted photons are calculated based on poissonian statistics. A defined level of poissonian autofluorescence background, optionally proportional to the local illumination power density, can be input. The program outputs the stack of acquisition frames, diffraction limited images, laser illumination profiles, and for each activated molecule, its *x* and *y* coordinates, fluorescence emission trace, the number of detected photons and an estimate of the signal-to-noise ratio.

Additional features, not of direct use for the current work, include possibilities to simulate dual-channel detection, two-color experiments (with two different fluorescent markers), sample drift, single-molecule Förster resonance energy transfer (FRET) between two fluorescent markers, and local activation/bleaching similar to what can be achieved with a Fluorescence Recovery After Photobleaching (FRAP) module.

### Biophysical model

**Figure 1:**
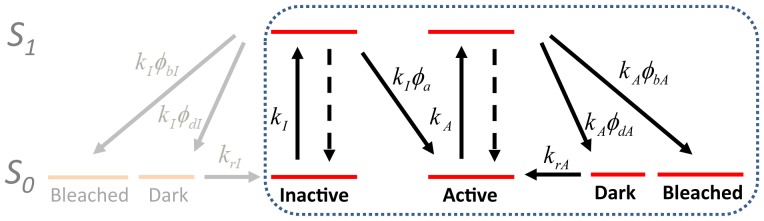
Photophysical model of Dendra2 phototransformations. The inactive state represents the neutral state of the green chromophore whereas the active state represents the anionic state of the photoconverted red chromophore. Fast exchange is assumed between protonated and neutral states of the chromophore. Possible bleaching pathways through the reversible dark state are neglected, as well as blinking and photobleaching pathways involving inactive molecules (in gray).

To extract the phototransformation yields of green-to-red photoconvertible fluorescent proteins, the photophysical model of Figure 1 is assumed. The inactive state of the marker corresponds to the green protonated chromophore whereas the active state corresponds to the red anionic chromophore. The green anionic chromophore (non-photoconvertible) and the red neutral chromophore (non-fluorescent) are thought not to play a role and are therefore not considered. However, rapid exchange between anionic and neutral chromophores relative to the acquisition frametime is assumed so that the rates of excitation in either the inactive or active states are evaluated based on extinction coefficients experimentally measured at a given pH.

Our photophysical model assumes that pathways leading to reversible dark states and to nonreversible bleached states are independent, similar to the approach taken by other groups [8], [33]. In doing so, we thus neglect the hypothesis that photobleaching may follow from photochemical transformation of a long-lived dark state. Although such an hypothesis has been proposed in several photophysical schemes [59], [60], we adopt this simplification based on our recent mechanistic investigations of IrisFP for which the major photobleaching pathways were suggested to differ from that leading to long-lived dark states [44].

In the model of Figure 1, the recovery from the dark to the active state is characterized by a single rate *k_rA_* and does not incorporate a quantum yield. This stems from the fact that in the absence of 405 nm light (readout activation mode) or in the presence of weak 405 nm light (typically used in PALM data acquisition), the process is essentially thermally induced (ref [8] and our own data (not shown)).

Finally, the model also assumes that blinking and bleaching following excitation of the inactive chromophore are negligible (Figure 1, left gray part). Such an assumption is justified because typical photoconversion yields measured to date are at least one order of magnitude higher than blinking and bleaching yields. Thus, the pool of inactive molecules competent for photoconversion is expected to be only marginally reduced at a given time. In addition, the various states of the inactive chromophore cannot be easily separated, as none of them fluoresces upon excitation by the readout laser.

### Experimental setup

A home-built PALM set-up was used based on an Olympus IX81 microscope (Olympus, Japan) equipped with diode-pumped solid-state 405 nm (CrystaLaser LC, USA), 488 nm (SpectraPhysics, USA) and 561 nm (Cobolt Jena, Sweden) lasers. Widefield illumination was achieved by focusing the laser beams to the back focal plane of a 60×1.45 *NA* oil immersion apochromatic objective lens (Olympus, Japan). The intensity and time-sequence of laser illumination at the sample was tuned by an acousto-optical tunable filter (AOTF, Quanta Tech, USA). Near circular polarization of the laser beams was ensured by inserting a polychromatic quarter-wave plate downstream the AOTF. To prevent sample drift during data acquisition, the samples were placed on an IX2-NPS “nosepiece” stage (Olympus) fixed directly on the objective. Fluorescence images were acquired with an Evolve back-illuminated EMCCD camera (Photometrics, USA) controlled by the Metamorph software (Molecular Devices, USA).

### Sample preparation

#### *In vitro* samples

Purified Dendra2 was diluted to nanomolar concentration in a pre-photobleached 1% solution of PVA in PBS at pH 7.4. Where appropriate, 1,4-diazabicyclo[2.2.2]octane (DABCO) (Sigma, USA) was added to a final concentration of 10 mM. The solution was spread as a thin film on a coverslip and let to harden at ambient temperature before PALM data acquisition. Control experiments with PVA devoid of Dendra2 showed no blinking single molecule (Figure S2).

#### *In cellulo* samples

Mammalian cells were grown on conventional coverslips or LabTek II 8-well chambered coverslips #1.5 (Nalgen-Nunc, USA). Before seeding the cells, LabTek chambers or coverslips were washed 3 times with 80% acetone in water, 100% methanol and milliQ water, immersed in 1 M solution of KOH overnight, and thoroughly washed with milliQ water. For expression of Dendra2-actin chimera in mammalian cells (HeLa Kyoto and Vero), we used the plasmid Dendra2-actin/pMC1 (described earlier [61]), which encodes for a humanized version of the Dendra2 gene from the pDendra2-C vector (Evrogen, Moscow, Russia). Cells were grown in complete Dulbecco's modified Eagle's medium (DMEM, Life technologies, USA) (4.5 g/L glucose, no phenol red) supplemented with 10% heat-inactivated fetal bovine serum (Life technologies), 2 mM glutamine, 100 µg/ml penicillin, 100 U/ml streptomycin and 1 mM sodium pyruvate (all from Life technologies), in 5% CO_2_ atmosphere at 37°C. No vitamin was added to the cell culture medium, which however already contains a complete set of vitamins, including riboflavin and pyridoxal [52], [62]. The cells were transfected with Dendra2-actin/pMC1 plasmids (0.5 µg/well) using Lipofectamine2000 (Invitrogen, USA) or FuGeneHD (Roche, Switzerland) as transfection reagents. At 24 to 48 hours post-transfection, the cells were washed with PBS, fixed with 4% paraformaldehyde (Sigma, USA) at 20°C for 30–60 min, washed with PBS 3 times, permeabilised with a 0.1% solution of Triton ×100 in PBS, and washed with PBS for another 3 times. Where appropriate, DABCO (Sigma, USA) was added to a final concentration of 10 mM. Cells were kept under pre-photobleached PBS at pH 7.4 during PALM data acquisition. The molecular oxygen level was in equilibrium with ambient atmosphere. The coverslips were mounted on microscopy slides using Parafilm gaskets and sealed with nail polish.

### Data acquisition

Reference laser beam profiles were recorded before each experiment using a coverslip uniformly marked with a fluorescent pencil. PALM datasets were acquired at room temperature (20–22°C) using two illumination schemes: 1) continuous illumination with 561 nm laser, which served both to photoconvert Dendra2 fluorophores and to readout the fluorescence of the activated red form; 2) continuous illumination with 561 nm and 405 nm laser. The power density at the focal plane was measured in each experiment and was typically ∼5–7 kW/cm^2^ at beam center for the 561 nm laser and ∼0.1–1 W/cm^2^ for the 405 nm laser. Data sets were acquired at 30 ms/frame.

### Processing pipeline

**Figure 2:**
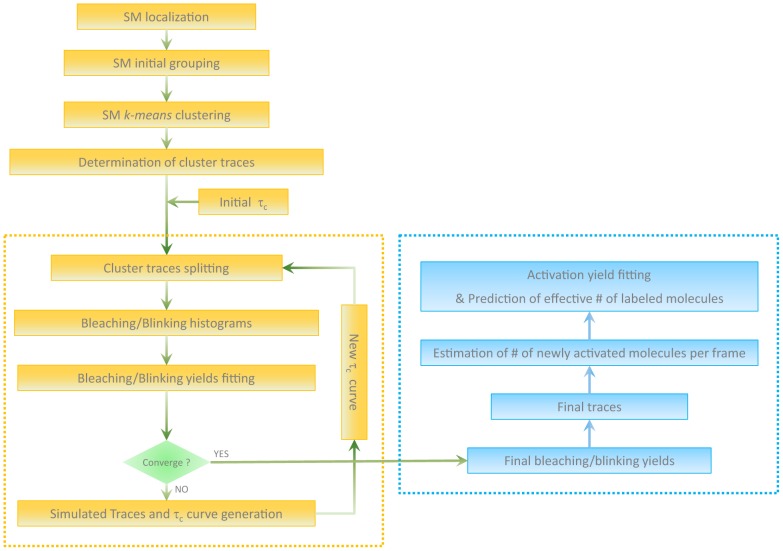
Sketch of the processing pipeline.

To determine the on-off blinking quantum yield, the off-on blinking rate, the photobleaching quantum yield as well as the photoconversion “brightness” (see below) of the fluorescent marker, the processing pipeline sketched in Figure 2 was developed in Matlab (Scripts S1).

Single-molecule localization is first performed based on standard 2-D Gaussian fitting of the diffraction-limited fluorescence spots, after rolling ball background subtraction. In addition to the precise localization of the fluorescent molecules, attention here is focused on extracting their accurate fluorescence traces. Indeed, removing a poorly localized spot from the dataset may result in introducing a glitch in a fluorescence trace, which would later be interpreted as a blinking event. Such problems may occur in the case of low signal-to-noise ratio or when multiple spots overlap at high molecular density. We modified our PALM localization software to carefully select spots judged appropriate for trace reconstruction (e.g. by setting criteria to allow center-of-mass based localization instead of rejection in the case of poor Gaussian fitting).

The next step consists of grouping localized spots found in subsequent frames, so as to constitute initial clusters of spots from which the most probably correct number of individual molecules and their associated fluorescence time traces can be extracted based on *τ_c_* splitting. To achieve initial clustering, localized spots are first connected in space and time according to coarse threshold criteria *d(s_1_,s_2_)<5σ*, where *d(s_1_,s_2_)* is the distance between spots *s_1_* and *s_2_* and *σ* is the localization uncertainty, and *t(s_1_,s_2_)<5τ* where *t(s_1_,s_2_)* is the time separation between spots *s_1_* and *s_2_* and *τ* is the maximum expected molecular lifetime. (We noticed that a more stringent distance criteria (e.g. 3*σ*) repeatedly leads to misclustering events.) However, such connection patterns can quickly lead to large groups of spots that spread over unrealistically large distances, especially in densely labeled and continuous biological structures. To subdivide such groups of spots into meaningful initial clusters, we developed an algorithm based on *kmeans* clustering analysis. Figure S3 shows an example of how densely packed localized spots are distributed into initial clusters.

Once initial clusters are determined, their fluorescence time traces can be built. We then use an approach similar to that introduced by Lee et al [8] to optimally estimate the number of molecules that constitute each cluster and extract their respective time traces. This approach consists of splitting cluster traces at every off-time *τ_off_* that is larger than a cutoff value *τ_c_*, so that the molecular counting error is minimized. The optimum *τ_c_* value has been shown to depend both on the blinking and bleaching rates of the fluorescent label and on the number *N* of labels present in the cluster [8]. As *N* is unknown, an iterative procedure was proposed, based on the computation of an optimal “*τ_c_*-curve”, *τ_c,opt_ = f(N)*, which was shown to rapidly converge to the correct number of molecules. Importantly, Lee *et al* construct their *τ_c_*-curve based on photophysical parameters of the fluorescent markers that are independently measured *in vitro*. In marked contrast, our goal is precisely to assess these parameters *in cellulo*. Thus, the following global iterative procedure was developed (Figure 2, orange dashed line). First, an initial fixed *τ_c_* value (e.g. 0.2 s) is used to split all cluster traces in the dataset so as to obtain a starting set of individual molecular fluorescence traces. These individual traces are then processed to extract bleaching and blinking histograms: *N_bleach_ = h(T_bleach_)*, *N_on_ = h(T_on_), N_off_ = h(T_off_)*, where *T_bleach_*, *T_on_* and *T_off_* represent the time before irreversible bleaching, blinking off, and blinking on, respectively, and *N_bleach_*, *N_on_* and *N_off_* represent the number of events within given ranges of *T_bleach_*, *T_on_* and *T_off_*. Based on our photophysical model (Figure 1), and on the proper evaluation of the spatially- and dipole-orientation-dependent molecular excitation rates (see below), these histograms can be fitted to extract a first evaluation of bleaching and *on-off* blinking quantum yields, as well as a rate for thermally activated *off-on* blinking. The self-consistency between these quantum yields can be verified by computing a blinking probability histogram, *P = h(N_blink_)*, where *N_blink_* represents the number of blinking events per molecule, and which is expected to follow a geometric distribution 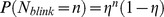 with 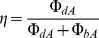 [8]. From the knowledge of Φ*_bA_*, Φ*_dA_*, *k_A_* and *k_rA_* it is then possible to generate a first *τ_c_*-curve, which is used to split again the cluster traces. The whole procedure is repeated until convergence, which typically requires less than 5 cycles. At the end of the process, final quantum yields and associated standard deviations are obtained, together with a quantitative evaluation of the total number of activated molecules. The number of newly activated molecules in each frame also becomes available, which allows for an estimation of the photoconversion brightness (see below) and the total number of (correctly matured) labeling molecules in the sample.

### Evaluation of excitation rates

To accurately fit bleaching and blinking histograms, a proper evaluation of the spatially- and dipole-orientation-dependent molecular excitation rate is necessary. The excitation rate [s^−1^] of a rapidly tumbling molecule located at position *(x,y)* is given by: 
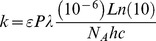
 where *ε* [M^−1^cm^−1^] is the extinction coefficient of the considered species (*I* or *A* molecules) at the excitation wavelength *λ* [nm], *P* [Wcm^−2^] is the laser power-density at *(x,y)*, *N_A_* is the Avogadro number, *h* is the Planck constant and *c* is the speed of light. In this work, chromophore extinction coefficients were derived from published reference values and fluorescence excitation or absorbance spectra at given pHs [61].

For a fixed oriented molecule with spherical coordinates *θ* and *φ*, the excitation rate of the absorbance dipole is angle dependent. For a circularly polarized laser beam whose electromagnetic field is assumed to be parallel to the objective focal plane, the excitation rate is given by: 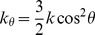, where *θ* is measured from the focal plane. For a linearly polarized laser beam whose electromagnetic field is in the objective focal plane and aligned with the *x* axis, the excitation rate becomes: 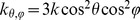 (see Appendix S1).

To handle the spatial heterogeneity of the laser beam under widefield illumination conditions, lasers operating in the TEM_00_ mode are assumed. In such a case, the intensity distribution is Gaussian, and experimentally recorded laser beam profiles can be fitted for their center *x_c_*, *y_c_* and their *1/e^2^* radii *r_x_*, *r_y_*. The power density at every single molecule positioned at *x_0_*, *y_0_* (taken as the average of the *x*, *y* coordinates of the localized spots constituting the molecule) is then obtained as:
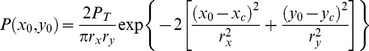
where *P_T_* is the total power measured at sample position. We note that the assumption of Gaussian beams is typically adequate in widefield illumination conditions, but not in Total Internal Reflection Fluorescence (TIRF) mode. In addition, the exponential decay of the electric field amplitude along the axial direction in TIRF mode and its complex polarization pattern would prevent a reliable evaluation of local excitation rates [63]. As a consequence, proper estimation of phototransformation yields is best achieved under widefield conditions, although at the expense of a lower signal-to-noise ratio.

In practice, to obtain reliable fits of the *N_bleach_* and *N_on_* histograms, molecules are sorted in a limited number of annular concentric subregions *R_1_*, *R_2_*, … *R_n_* in which *P(x,y)* is considered homogeneous with average power density 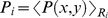. Global fits of the photobleaching and on-off blinking quantum yields are then performed according to the following formula:
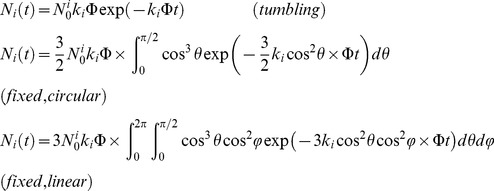
Obtaining the thermally activated rate for return from the dark state, *k_rA_*, is simpler because there is no dependence on chromophore position or dipole orientation: assuming a homogeneous population of molecules: 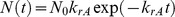.

### Determination of photoconversion brightness under readout laser illumination

As already mentioned, once optimal splitting of the cluster's traces is achieved, the number of newly activated molecules per frame along data collection can be extracted. In the presence of readout excitation only, and assuming constant illumination during the acquisition time, the green-to-red photoconversion yield Φ*_a_* as well as the total number of labeled molecules present in the investigated sample area can in principle be obtained. In the case of rapidly tumbling molecules, the accumulated number of activated molecules along time *N_A_(t)* can be fitted according to:
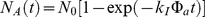
where *N_0_* is the total number of labeled molecules and *k_I_* is the excitation rate of inactive molecules by the readout beam. However, a difficulty is that it is practically impossible to accurately estimate the extinction coefficient of the inactive molecules (green protonated chromophores) at the readout excitation wavelength (typically 561 nm), *ε_561_* (Figure S3). This stems from the fact that, in this wavelength region, the absorbance spectrum of protonated green molecules (which exhibits a broad band centered at ∼400 nm) cannot be reliably measured, even at very low pH, because not only is it very weak but also it is always obscured by residual overlapping absorbance from anionic chromophores. One solution to get around this problem is to report the photoconversion brightness, instead of the photoconversion quantum yield, defined as *φ_a_* = *ε*_561_Φ*_a_*. Indeed, the quantity that can be accurately fitted is *k_I_*Φ*_a_*, and thus the photoconversion brightness is given by *φ_a_* = 5.21×10^4^ *k_I_*Φ*_a_*/*Pλ*. Overall, similarly to the fitting procedure for retrieving photobleaching and on-off blinking quantum yields, the photoconversion brightness and total number of labeled molecules are obtained according to the following formula:
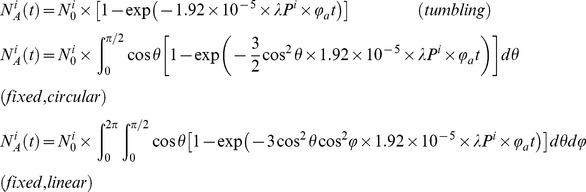



### Determination of photoconversion quantum yield under 405 nm activation laser

In the presence of weak 405 nm activation, the same procedure as described above can be used as long as the density of activated molecules in each frame can be managed. An apparent photoconversion brightness *φ_a,app_* can then be extracted (which results from photoconversion by both activation and readout lasers), from which the photoconversion quantum yield at 405 nm Φ*_a_*_,405_ can be estimated as: 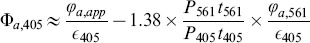, where *P_561_* and *P_405_* refer to the power densities at beam center, *t_561_* and *t_405_* refer to exposure times and 1.38 is the ratio of the two wavelengths.

## Results

### Simulated data

To test the proposed strategy for estimating phototransformation yields, we first simulated a representative PALM data set using our Matlab-based simulation software. 30000 Dendra2 molecules were randomly spread over a microtubule-mimicking pattern (corresponding to a molecular density of 545 molecules/µm^2^) and 6000 frames with 30 ms exposure were generated using circularly polarized 561 nm laser excitation. No 405 nm activation laser was used and, under readout laser activation, the green-to-red photoconversion brightness was set to 4.3×10^−4^ M^−1^cm^−1^ (Φ*_a_* = 1.2×10^−4^; *ε_561_* = 3.57 M^−1^cm^−1^), so that the average density of activated molecules per frame amounted to 0.22/µm^2^. The simulated sample was assumed to be representative of a live cell, so that each Dendra2 molecule was modeled as freely tumbling. Input values for the bleaching yield, the blinking-off yield and the thermally activated blinking-on rate were taken as 2×10^−5^, 5×10^−6^ and 20 s^−1^ respectively, close to the experimental values that can be extracted from Lee *et al* [8]. A complete list of parameters used to generate this PALM dataset is shown in Table S1. The simulated sample is shown in Figure S1.

The dataset was then submitted to the processing pipeline described above. Erroneous phototransformation yields and blinking-on rate were deliberately input to the fitting routines as starting values (with deviations by an order of magnitude relative to true values). Five concentric regions where the laser beam can be considered homogeneous were defined as shown in Figure S5. Convergence was achieved after 3 cycles and the final retrieved values are reported in Table S2 and compared with the “true” values used in the simulation. A representative *τ_c_*-curve is shown in Figure S6 and histograms of the correlation coefficients between true and retrieved single-molecule traces are presented in Figure S7.

The photobleaching yield was retrieved with high-fidelity, with an error less than 1%. Recovery of the blink-on rate was also satisfactory (21.9 s^−1^, 9% error). However, a substantial discrepancy was obtained between the retrieved and the true blink-off yields. The retrieved yield (3.6×10^−6^) was smaller than the true yield, with an error of 28%. This finding is explained by the limited time-resolution of PALM data acquisition (see Discussion S1, Figure S8, and see below). Retrieval of the photoconversion brightness was fully satisfactory (4% error), allowing at the same time to extract an accurate estimation of the number of labeled molecules present in the sample (8% error), a crucial aspect in quantitative super-resolution microscopy [9], although this retrieved number does not take into account additional effects such as reporter misfolding or presence of a non-photoconvertible fraction of the labels [7], [21].

### Effect of dipole orientations and laser polarization

Two additional mock data sets were generated using the same set of parameters except that this time the simulated sample was assumed to be representative of a fixed cell: each Dendra2 molecule was assigned a random dipole orientation, constant over time. In the first dataset a circular polarization of the laser beam was assumed whereas a linear polarization was chosen in the second one. In both cases, using the appropriate fitting models, the quality of yield extraction followed the same trend as for tumbling molecules, although the overall accuracy was slightly lower for circular polarization and significantly lower for linear polarization (Table S3). This deterioration follows from the strong fluctuation in the ability of each molecule to absorb photons. The results were further degraded if a fitting model corresponding to tumbling molecules was used. This can be understood by inspection of Figure S9: in the case of molecules with fixed orientations and a circularly polarized laser, a few molecules experience a higher excitation rate than the average rate experienced by a tumbling molecule (i.e. due to an absorbance dipole parallel to the objective focal plane) or a lower rate (i.e. due to an absorbance dipole along the optical axis). As a consequence, these molecules systematically exhibit shorter or longer fluorescence time traces, slightly distorting the shape of the *N_bleach_* and *N_on_* histograms. In the case of a linearly polarized laser, this distortion is more pronounced (Figure S9). Likewise, the shape of the cumulative plot of the number of photoconverted molecules along time is distorted when molecules adopt fixed orientations, as compared to tumbling molecules, and more so when the excitation laser is linearly polarized (Figure S9).

These simulated data highlight the importance of taking into account fluorophore dipole orientations in such studies. Nevertheless, based on the results presented in Table S3, we anticipate that, at least when circularly polarized lasers are used, errors made by assuming a somewhat incorrect molecular orientation model (as could be the case in biological samples) should be relatively small in comparison with other sources of errors (see below).

### Retrieval of blinking-off yield

**Figure 3:**
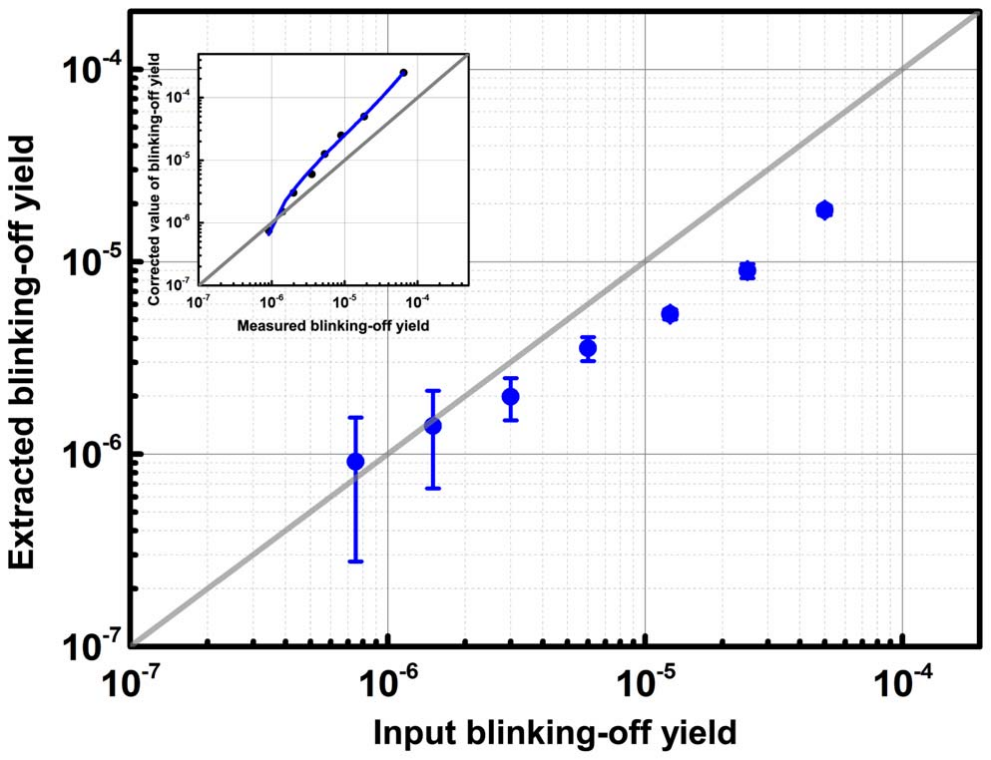
Accuracy of blinking-off quantum yield retrieval. Extracted blinking-off quantum yields are plotted against a range of input yields used in a series of simulated PALM data sets. Those simulated data sets were generated using parameters displayed in Table S1 (except for the varying blinking off input yield). Error bars represent fitting errors. Yield retrieval for highly blinking molecules underestimates the true values, due to the limited time-resolution in PALM experiments. For weakly blinking molecules, retrieval accuracy is superior, but precision is worsened (larger error bars) because of statistical uncertainty due to low numbers of blinking molecules. The inset shows the same curve in an inverted manner. A polynomial fit to the data points provides a potential calibration curve allowing, in real experiments, to estimate the true yield from the extracted yield.

The noticeable underestimation of the blinking-off quantum yield in the above simulations calls for an investigation of the range of blinking yields that can be adequately retrieved under typical PALM data acquisition parameters. To this aim, we performed a number of simulations in which the blinking-off quantum yield was systematically varied, assuming a fixed cell with circularly polarized laser, and keeping all other parameters identical to those described above. The results (Figure 3) show that retrieval of the blinking-off yield progressively deviates from (underestimates) the true value as the latter is increased. This is explained by the fact that, as the input yield is increased, more and more short on-times events are missed due to the limited time-resolution of the experiment (see Discussion S1 and Figure S8). On the other hand, for low yields on the order of 1.0×10^−6^ (or rather 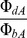
<<1), a significant statistical uncertainty is observed, due to the fact that very few molecules experience blinking, thus giving rise to noisy *N_on_* histograms. Interestingly, Figure 3 suggests that in the range ∼2.0×10^−6^-1.0×10^−4^ an accurate determination of the correct yield could be retrieved if a proper calibration is done. Calibration curves of the type shown in Figure 3 may be obtained from simulated data in which experimental parameters are reproduced faithfully, notably the rate of excitation of the fluorescent molecules and the acquisition frametime. Finally, Figure S11 shows that the correct extraction of the bleaching quantum yield, photoconversion brightness and off-on blinking rate are not significantly affected by the value of the blinking-off yield, except at very high blinking yield >1.0×10^−4^.

### Evaluation of suitable experimental parameters

**Figure 4:**
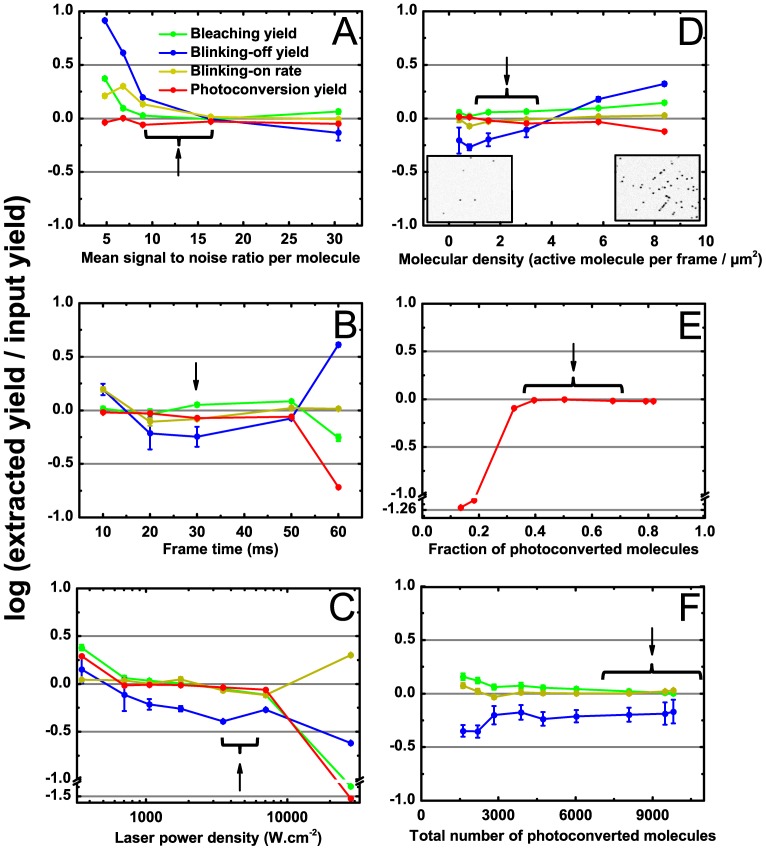
Effect of experimental parameters on accuracy of phototransformation yield's retrieval. In each panel, extracted phototransformation yields are plotted against a key experimental parameter (signal to noise ratio per molecule (**A**), frametime (**B**), laser power (**C**), molecular density per frame (**D**), fraction of photoconverted molecules (**E**) and total number of photoconverted molecules (**F**)). In each case, PALM datasets were simulated using parameters of Table S1 (except for the varying parameter). Black: bleaching yield, blue: blinking-off yield, gray: blinking-on rate, red: photoconversion yield. Yield retrieval accuracy is estimated by the logarithm of the ratio between true and extracted yields and error bars represent fitting errors (with 95% confidence). In **D**, the insets show representative simulated frames at molecular densities of 1 and 8 active molecules per frame per square micron, respectively. In **E**, only the extracted photoconversion yield is shown, as the input parameter is not relevant to bleaching and blinking. In **F**, conversely, only extracted bleaching and blinking yields are represented. Experimental data discussed in this work have been obtained within ranges of conditions indicated by braces and/or black arrows. Molecular densities per frame cannot be easily assessed for experimental data and therefore reported values are only indicative.

In order to estimate experimental conditions suitable for accurate determination of phototransformation yields, a number of additional simulations were performed in which the same set of yields (representative of Dendra2) were input and the following key parameters were systematically varied: background level, frametime, laser power, molecular density and number of frames. The results are presented in Figure 4.

#### Background level

High autofluorescence or background noise due to out-of-focus light is a serious concern when cellular samples are investigated using a widefield geometry. Such noise may prevent the detection of many fluorescent spots, which results in a significant number of single-molecule traces being chopped into shorter traces. This in turn translates into increased apparent bleaching and blinking yields. At medium signal-to-noise ratio (*SNR*, defined as the photon output of individual spots divided by the standard deviation of the background over the spot area), this effect actually compensates for the underestimation resulting from time truncation (see above), explaining why errors are not at their minimum in the absence of background noise (Figure 4A). Importantly, *in vitro* data reported in this work typically match this medium signal-to-noise ratio (*SNR* ∼15), whereas *in cellulo* data are slightly worse (*SNR* ∼10), in a regime where phototransformation yields could be slightly overestimated.

#### Frametime

reducing the frametime enables enhanced time resolution at the expense of reduced signal-to-noise ratio. Inspection of Figure 4B reveals that, given the input value we have chosen for the yields, a good compromise is maintained in the range 10–30 ms frametime. At the shortest frametime, the reduced *SNR* results in a slight overestimation of the photoblinking regime, whereas at the longest frametime (60 ms) most fluorescence time traces are contained within a single frame, completely obviating the ability to recover correct yields.

#### Readout laser power density

Figure 4C shows that variation of the laser power density within the range 0.2–20 kW/cm^2^ qualitatively has the same effect as changing the frametime. This can be easily understood, as, at low laser power, reduced *SNR* results in yield's overestimation, whereas at high laser power fluorescence traces get shorter due to increased photoexcitation, giving rise to insufficient time resolution.

#### Molecular density

In contrast to *in vitro* samples, where a sufficiently sparse distribution of immobilized fluorescent molecules can be achieved to prevent overlap of fluorescence traces, in biological samples fluorescent labels typically decorate a fine structure of interest and their local density can be very high. This translates into a substantial density of active molecules per frame. Figure 4D shows that extraction of phototransformation yields remains successful at densities up to 7 molecules/frame/µm^2^ on our microtubule mimicking pattern (note that such densities on an essentially 1-D pattern cannot be directly compared to densities extracted from 2-D patterns [57]). At very high densities, an overestimation of the yields is observed, which results from rejection of strongly overlapped spots and heavy mix up of a large number of fluorescence traces (Figure 4D, inset). However, the results suggest that typical expression levels in biological cells do not need to be reduced to retrieve correct photophysical parameters, at least under weak 405 nm laser illumination.

#### Number of frames

Figure 4D shows that retrieval of the correct photoconversion brightness necessitates a substantial fraction of the total number of molecules to be photoconverted during the experiment (>30%). This is explained by inspecting the exponential dependence of *N_A_*(*t*) = *N*_0_[1−exp(−*αφ_a_t*)] (see also Figure S10). As long as *N_A_*(*t*) can be approximated by a linear regime *N_A_*(*t*)≈*N*_0_ *αφ_a_t* (*αφ_a_t*<<1, *α* is a constant) the unknown parameters *N_0_* and *φ_a_* cannot be uncoupled and unreliable values are extracted. Finally, Figure 4F simply shows that for a fixed sample and in the presence of a heterogeneous laser beam a substantial number of activated molecules need to be recorded so that sufficient statistics allow reliable extraction of the phototransformation yields.

Overall, the analysis of simulated datasets presented above demonstrates that our procedure estimates the phototransformation yields with suitable accuracy and robustness in a large range of experimental parameters. This constituted the starting point for choosing appropriate settings for real experiments and estimating the suitability of experimental data sets for analysis.

### Experimental data

#### *In vitro* data

**Figure 5:**
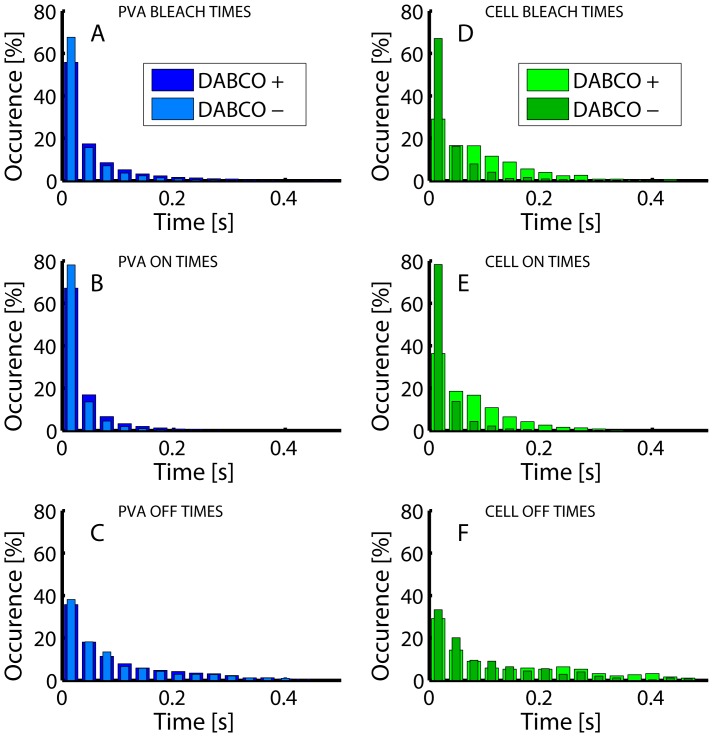
Representative histograms of *N_bleach_*, *N_on_*, *N_off_* from *in vitro* and *in vivo* Dendra2 photophysics. **A–C**: PVA-embedded Dendra2 in the absence and in the presence of 10 mM DABCO; **D–F**: Fixed Vero cells expressing Dendra2-actin, in the absence and in the presence of 10 mM DABCO.

**Figure 6:**
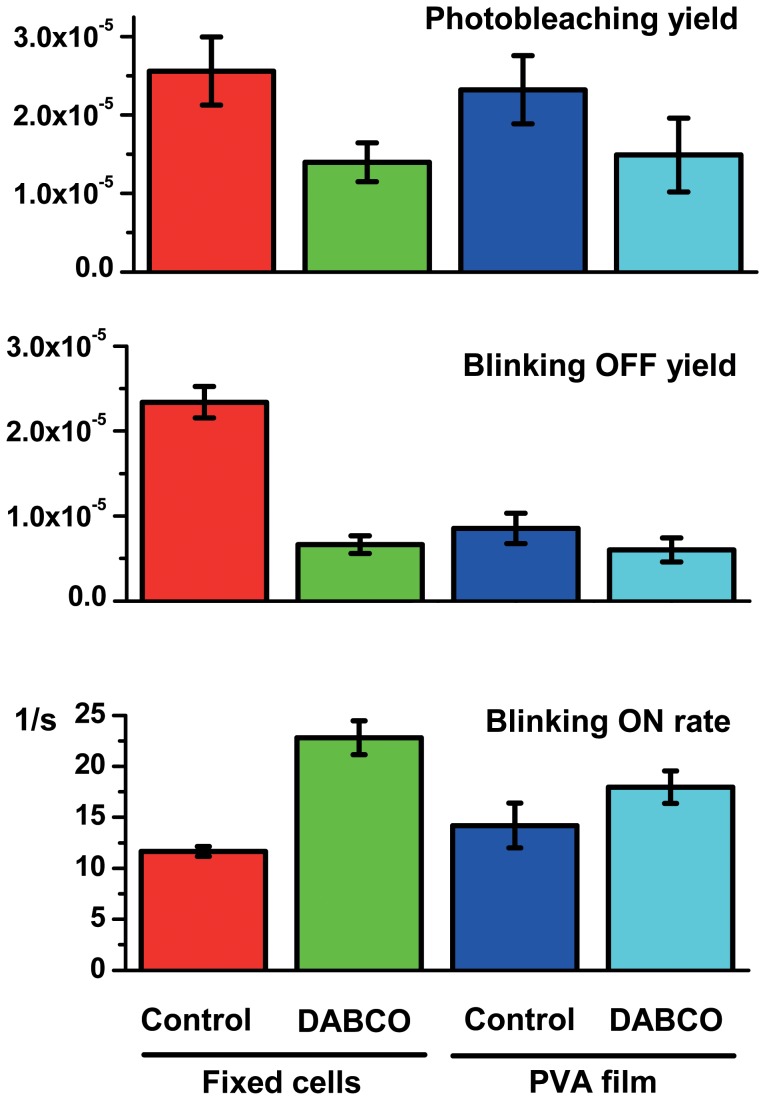
Phototransformation yields of Dendra2-actin expressed in mammalian cells and purified Dendra2 embedded into PVA film. Error bars indicate standard deviations. Number of samples per experiment *n* = 3–5. Cell samples are from different batches prepared independently.

**Figure 7:**
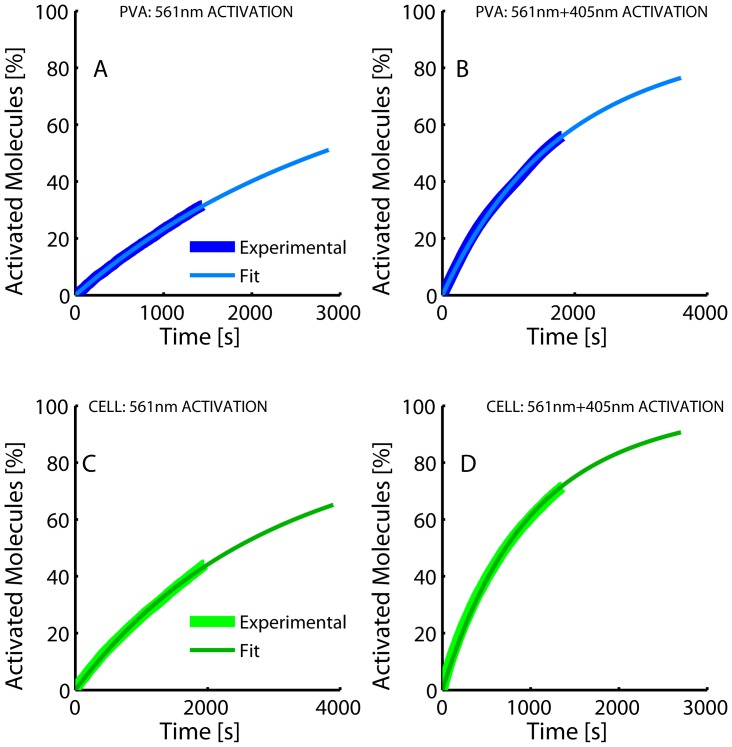
Representative cumulative photoconversion plots of purified Dendra2 embedded into PVA film (A, B) and Dendra2-actin expressed in mammalian cells (C, D); (illumination with 561 nm laser only (A,C) or in combination with 405 nm laser (B, D). Fitted curves are shown overlaid on experimental data.

The phototransformation yields of Dendra2 were first measured *in vitro* in PVA, under conditions of typical PALM data acquisition, with or without 405 nm activation (see Data acquisition section and Figure S2). The processing pipeline with the “tumbling” model was used. Results are reported in Figures 5 to 7. The obtained values are close to those that can be deduced from the phototransformation rates reported by Lee et al [8], although a different immobilization technique was used. With bleaching and blinking-off quantum yields of (2.3±0.4)×10^−5^ and (8.6±1.8)×10^−6^, respectively, Dendra2 exhibits a fair photostability and has a relatively low tendency to blink, with on average one out of 3.7 molecules experiencing blinking. The rate of recovery from the dark state was measured to be 14±2 s^−1^, corresponding to an average lifetime in the off state of 70 ms. Dark state recovery is essentially thermally driven and was found to be accelerated by 405 nm light only at laser intensities greater than (∼10 W/cm^2^), which exceeds the values typically used in PALM acquisition, as previously demonstrated [8] (not shown). Likewise, blinking and bleaching quantum yields were not modified upon weak 405 nm laser light illumination. Upon treatment with 1,4-diazabicyclo[2.2.2]octane (DABCO), an antifading agent commonly used in fluorescence microscopy to reduce photobleaching [64], [65], [66], the photobleaching and blinking-off yields slightly decreased (*Φ_bleach_*: (1.5±0.5)×10^−5^; *Φ_blink-off_*: (6.0±1.4)×10^−6^), while the blinking-on rate slightly increased (*Φ_blink-on_*: 18±1.6 s^−1^), but all these changes were not significant (Figure 6).

Finally, from the analysis of the cumulated number of photoconverted molecules over the time of PALM data acquisition (Figure 7), the photoconversion brightness of Dendra2 was measured to be (0.9±0.2)×10^−5^ M^−1^cm^−1^ under 561 nm readout laser illumination. As expected, the brightness measured under 405 nm illumination was substantially higher ((4.1±2.3)×10^−1^ M^−1^cm^−1^) and, assuming an extinction coefficient of 10250 M^−1^cm^−1^ at pH 7.4, as measured by ensemble spectroscopy, a surprisingly low photoconversion quantum yield of (4.0±2.3)×10^−5^ could be deduced. Results are summarized in Table 1. Based on the coarse assumption of a wavelength independent quantum yield, the residual extinction coefficient of the neutral green state of the Dendra2 chromophore could be proposed to be ∼1.0 M^−1^cm^−1^ at 561 nm at pH 7.4. However, this statement would not hold if photoconversion in the anionic green state of Dendra2 also occurs as recently suggested [67].

**Table 1:** Photoconversion parameters of Dendra2.

	PVA film	Mammalian cells
**Photoconversion brightness (561 nm) [M^−1^cm^−1^]**	(0.9±0.2)×10^−5^	(0.9±0.4)×10^−5^
**Photoconversion brightness (405 nm) [M^−1^cm^−1^]** $	(4.1±2.3)×10^−1^	(1.4±0.6)×10^−1^
**Photoconversion yield (405 nm)** $ #	(4.0±2.3)×10^−5^	(1.4±0.6)×10^−5^

Mean values ± SD are shown. Number of samples per experiment n = 3.

$ : The high SDs are attributed to inhomogeneity and weakness of the 405 nm laser beam.

# : Assuming an extinction coefficient *ε_405_* = 10250 Mol^−1^ cm^−1^.

### Eukaryotic cells

Next, we measured the phototransformation yields of Dendra2 fused to actin in fixed mammalian Vero cells. A representative PALM rendered image is shown in Figure S12 and the results of analysis are shown on Figures 5 to 7. The processing pipeline with the “fixed-circular” model was used. Importantly, experimental conditions (notably signal-to-noise ratio) were found to fall within the range suitable for accurate extraction of phototransformation yields (Figure 4). A photobleaching quantum yield of (2.6±0.4)×10^−5^ was measured, close to the value obtained in PVA. In marked contrast, the blinking-off quantum yield (2.3±0.2)×10^−5^ was found to largely exceed that measured *in vitro*, whereas the blinking-on rate (11.7±0.5 s^−1^) was slightly lower. Consequently, about half of the chromophores experienced blinking, a fraction significantly higher than in PVA, and also higher than the 30% ratio reported in *Xenopus* oocytes [21].

Strikingly, and in marked contrast with PVA, DABCO induced a significant enhancement of Dendra2 photostability in fixed Vero and HeLa cells. The photobleaching quantum yield was reduced nearly 2-fold ((1.4±0.2)×10^−5^), the blinking-off quantum yield was decreased more than 3-fold ((6.6±1.0)×10^−6^), whereas the blinking-on rate was increased by nearly 2-fold (22.8±1.7 s^−1^). In fact, adding DABCO to the PBS medium used for cell imaging brought the phototransformation yields of Dendra2 close to those measured in PVA alone and nearly identical to those measured in PVA plus DABCO. However, DABCO did not cause an increase of mean photon output per molecule, possibly in agreement with the previously reported quenching effect of this substance [65].

Finally, the green-to-red *in cellulo* photoconversion brightness of Dendra2 was measured to be (0.9±0.4)×10^−6^ M^−1^cm^−1^ and (1.4±0.6)×10^−1^ M^−1^cm^−1^, under 561 nm and 405 nm illumination respectively (Figure 7). In the latter case, assuming an extinction coefficient of the neutral form of green Dendra2 at 405 nm of 10250 M^−1^cm^−1^ [36], a photoconversion yield of ∼(1.4±0.6)×10^−5^ could be deduced, even lower than in PVA (Table 1).

## Discussion and Conclusions

In this work, we have developed a reliable method to evaluate *in cellulo* the photo-transformation yields of fluorescent proteins used as markers in high-resolution localization microscopy. Through extensive simulations, we have determined the range of experimental parameters suitable for accurate determination of the photobleaching yield, the blinking-off yield, the blinking-on rate as well as the photoconversion brightness of PTFPs. The method was then applied to study the *in cellulo* photophysical behavior of Dendra2 and the results were compared to those obtained in PVA. An important conclusion is that the photophysical behaviors of fluorescent proteins measured *in vitro* and *in cellulo* may differ. Quantitative localization microscopy therefore would benefit from a preliminary investigation of the fluorescent marker's bleaching and blinking behavior in biologically relevant experimental conditions. In the case of Dendra2 fused to β-actin in fixed mammalian cells at physiological pH, we found a bleaching yield of (2.6±0.4)×10^−5^, a blinking-off yield of (2.3±0.2)×10^−5^ and a thermally activated blinking-on rate of 11.7±0.5 s^−1^. Interestingly, when the antifading agent DABCO was added at 10 mM to the PBS-based medium in which mammalian cells were mounted, the photostability of Dendra2 was largely enhanced. Reversible blinking was decreased more than 3-fold and nonreversible photobleaching was reduced by about 2-fold. Furthermore, the average duration of Dendra2 transient dark states was reduced from ∼85 ms to ∼45 ms. Thus, because less molecules will blink, and those that still blink will more rapidly return from their dark state, the use of DABCO in combination with Dendra2 may facilitate accurate molecular counting in quantitative PALM.

While redox agents and oxygen scavenging systems have been carefully evaluated in STORM/dSTORM localization microscopy to ensure optimal blinking of the organic labels [47], the chromophores of fluorescent proteins are often considered insensitive to the local environment and FP-labeled samples are commonly prepared without any additives. However, recent data clearly demonstrated that the photophysical properties of fluorescent proteins are sensitive to the environment [50], [52], [68], [69]. Our results go along this line.

Two main scenarios can be invoked to account for the observations reported in Figure 6. Firstly, DABCO is known to retard photobleaching of various fluorophores in immunofluorescence samples, which was explained by hampering diffusion of oxygen and other reactive oxygen species (ROS) capable of destroying the fluorophore [64], [65], [66]. In addition, PVA is known to also hamper diffusion of oxygen [4]. Thus, a reduced oxygen level could possibly explain the enhanced photostability in PVA or in DABCO-enriched PBS if blinking and bleaching mechanisms under PALM experimental conditions are oxygen dependent. Alternatively, DABCO is also an electron acceptor and it has been shown that PVA may be involved in electron-transfer processes to/from organic dyes in their excited states, thereby affecting the blinking properties of these dyes [70], [71]. Thus, our results could as well be explained by redox effects.

Our previous investigations of IrisFP, a PTFP which shares high sequence-homology and structural-similarity with Dendra2 [42], [43], [44], lead us to suggest that this latter scenario predominates. Under high-intensity illumination as typically used in localization microscopy (>0.1 kW/cm^2^) structural studies indeed revealed that IrisFP photobleaching essentially follows a redox-driven process associated with the decarboxylation of the conserved Glu212 residue (IrisFP numbering) [44]. This photodestruction pathway leads to a non-reversible 2-electron photoreduction of the chromophore, likely to be modulated by the local redox environment. The process is oxygen-independent, in contrast to the photobleaching pathway observed at lower illumination levels typical of standard widefield microscopy. Likewise, our investigations of blinking in IrisFP [42], [43] also suggested a redox process in which the FP chromophore undergoes a transient loss of electron conjugation at the methylene bridge, followed by proton transfer. DABCO and PVA could play a role in such mechanisms. Further investigations will need to be carried out to confirm or infirm this hypothesis. Preliminary *in cellulo* experiments performed under anaerobic conditions suggest that indeed the measured Dendra2 phototransformation yields do not depend on oxygen significantly.

More generally, it will be interesting in the future to carry out more systematic studies of the effects of DABCO and other antifading agents such as e.g. trolox, on photoblinking and photobleaching of various fluorescent proteins. In addition, in the present study we have not looked at a possible dependence on DABCO of the Dendra2 photoconversion brightness. It will be informative in future experiments to investigate the possible redox dependence of green-to-red photoconversion in various PCFPs, as reported for example in mEos2 [51].

The phototransformation yields in Dendra2 reported in this work fall within the range of values determined for other PTFPs by various ensemble or single molecule methods. Although yields measured in the exact conditions of biological PALM experiments are expected to be most informative, their accuracy could be affected by various complications not accounted for in the present work, such as local heterogeneity of the laser beam, nonuniform background, highly anisotropic distribution of PTFP's dipole orientations, or misestimation of laser power density due to optical aberrations. Importantly, extinction coefficients are not measured *in vivo*, are pH dependent and are difficult to estimate accurately. Reliable measurements of phototransformation quantum yields require finding a good compromise between high signal-to-noise ratio of the recorded fluorescence and sufficient time-resolution to faithfully reconstruct single-molecule traces. In particular, we have shown that the limited time-resolution of PALM experiments may significantly bias the retrieval of blinking-off quantum yields, although such bias could possibly be corrected by generating calibration curves from simulated data. For example, in the case of the fixed Vero cells, applying the calibration curve of Figure 3 (inset) would suggest a blinking-off quantum yields of (6.7±0.6)×10^−5^ and (1.5±0.1)×10^−5^ in the absence and presence of DABCO, respectively, meaning that DABCO induces a drop in blinking by a factor ∼5. However, one should bear in mind that a proper calibration needs to faithfully account for a high number of experimental parameters, and we propose that more thorough investigations should be conducted before corrections can be confidently applied. Perhaps even more important, cell to cell variability always remains an issue to take into account and it cannot be excluded that sample preparation protocols as well as specific experimental conditions (e.g. temperature or presence of vitamins in the cell culture medium [52]) may exert an influence on the extracted phototransformation yields. Therefore well-defined artificial environments with single-molecule or even bulk measurements will continue to provide valuable reference values that should not be overlooked.

Within all these limitations, our extensive simulations suggest that accurate phototransformation quantum yields can be retrieved if they fall within reasonable ranges and that reliable comparative studies between different samples or various environmental conditions can be conducted.

Within the precision of our measurements, Dendra2 molecules displayed a homogeneous photophysical behavior, as *N_bleach_*, *N_on_* and *N_off_* histograms could be satisfactorily fit with mono-exponential models. This is in contrast with other findings made *in vitro* where recovery to the fluorescent state exhibited a biphasic behavior [8], the mechanistic interpretation of which remains unclear. Our data suggest that various conformational states of Dendra2, if they exist, interconvert at rates that exceed the measured macroscopic rates.

Our simplified photophysical scheme (Figure 1) may also be refined in the future to extract more accurate phototransformation yields. With more statistically significant data, multiphasic bleaching and blinking processes could be pinpointed [72], and the possibility that bleaching may directly follow from blinking [59], [60] could be investigated. One observation is that the recovered green-to-red photoconversion brightness (or yields) for Dendra2 appears lower than expected based on the few reported bulk experiments on this protein [67] and other PCFPs [18], [19]. The photoconversion quantum yields measured here under 405 nm activation are of the same order of magnitude as the photobleaching or blinking-off quantum yields. This calls for an experimental strategy in which bleaching and blinking in the green state of the fluorophores are taken into account, as well as further photophysical processes that may be responsible for the generally observed limited overall photoconversion efficiency, as recently shown [21].

Finally, the present study opens the door to a more systematic investigation of the influence of environmental parameters on the photophysical behavior of markers or interest for single-molecule localization microscopy. The technique may for example be extended to the case of organic dyes [73], allowing a precise assessment of the effects of various blinking cocktails used in techniques such as dSTORM.

## Supporting Information

Figure S1 **View of our Matlab-based PALM simulation software.**
(PDF)

Figure S2 **Representative frames from a PALM dataset of a “blank” PVA sample devoid of Dendra2 molecules (left) and a PVA sample containing Dendra2 (right).**
(PDF)

Figure S3 **Representative example of spot's clustering by the** ***k-means*** **algorithm.**
(PDF)

Figure S4 **Absorption spectrum of Dendra2 in its green state collected at acidic pH, after deconvolution of a residual contribution by the chromophore in its anionic state.**
(PDF)

Figure S5 **Rendered PALM image of the simulated microtubule sample (top) and 2D profile of the simulated laser beam (bottom).**
(PDF)

Figure S6 **Final** ***τ_c_*** **-curve extracted from a PALM data set generated with the parameters shown in Table S1.**
(PDF)

Figure S7 **(A) Representative example of single-molecule trace reconstruction.** (B) correlation histogram between the retrieved single-molecule traces and true simulated traces. (C) correlation histogram between the retrieved single-molecule traces and rounded true simulated traces.
(PDF)

Figure S8 **Effects of limited time-resolution on the determination of photo transformation yields.**
(PDF)

Figure S9 **Representative** ***N_bleach_*** **histograms for tumbling molecules (A), fixed- molecules under circularly polarized laser (B), and fixed molecules under linearly polarized laser (C).**
(PDF)

Figure S10 **Plots of cumulative activation for tumbling molecules (A), fixed molecules under circularly polarized laser (B), and fixed molecules under linearly polarized laser (C).**
(PDF)

Figure S11 **Errors in retrieved photobleaching yield,** ***on-off*** **blinking rate and photoconversion yield (or brightness) upon varying the input** ***off-on*** **blinking yield.**
(PDF)

Figure S12 **Rendered PALM image of Dendra2-β-actin in a fixed HeLa cell under widefield illumination conditions.**
(PDF)

Appendix S1 **Excitation rate of fixed molecules.**
(PDF)

Discussion S1 **Underestimation of blinking-off quantum yield.**
(PDF)

Scripts S1 **Matlab scripts for simulation of PALM data sets and for the extraction of phototransformation yields.**
(ZIP)

Table S1 Parameters used to generate simulated PALM data sets.
(PDF)

Table S2 Comparison between phototransformation yields retrieved from true single-molecule traces, rounded true traces and experimental traces.
(PDF)

Table S3 Comparison between phototransformation yields retrieved from simulated data sets with tumbling molecules and fixed molecules.
(PDF)
