# Supplementary material for: In cellulo Evaluation of Phototransformation Quantum Yields in Fluorescent Proteins Used As Markers for Single-Molecule Localization Microscopy
Source: PLoS One. 2014 Jun 10;9(6):e98362. doi: 10.1371/journal.pone.0098362 (PMC4051587; doi:10.1371/journal.pone.0098362)
Supplement: Figure S1 — View of our Matlab-based PALM simulation software. (PDF) [file pone.0098362.s001.pdf]

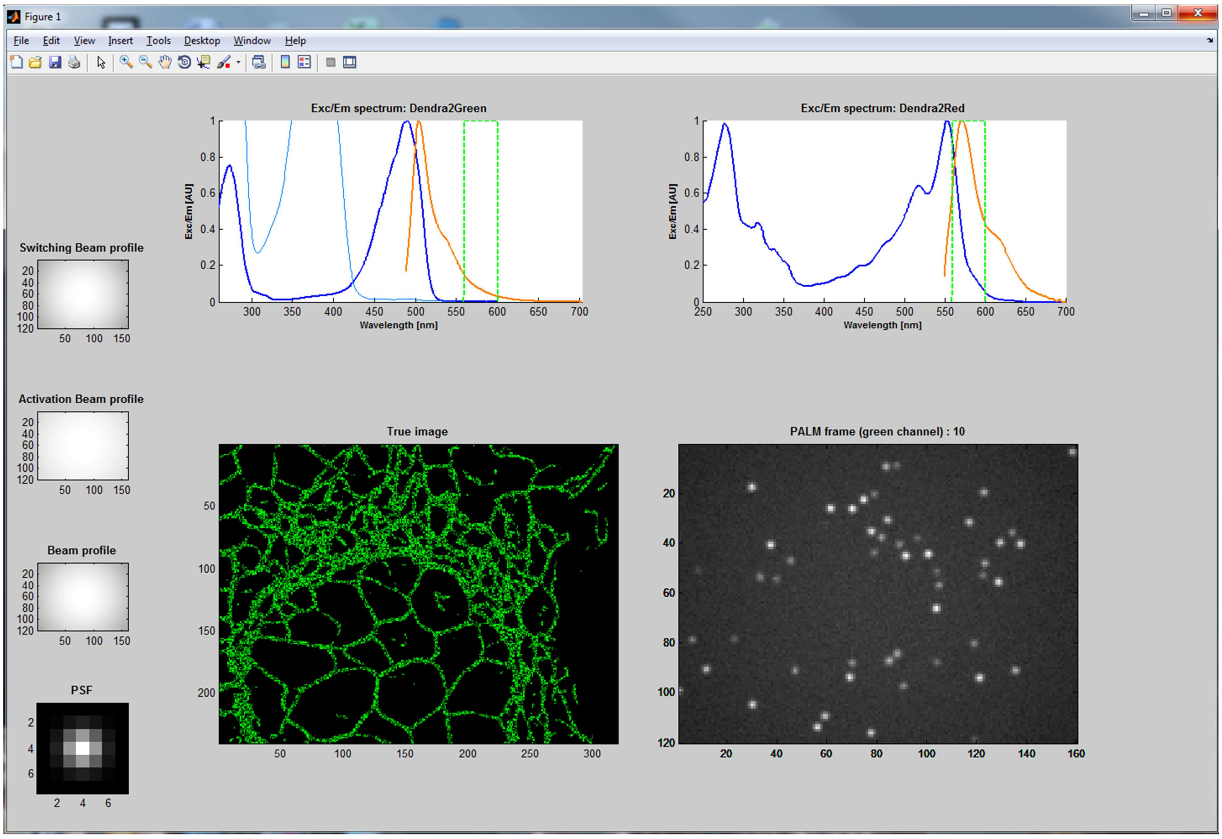

Figure S1: View of our Matlab-based PALM simulation software. The microtubule pattern used to generate the data sets is shown in green. Spectroscopy data characterizing the used PTFP are shown on the top, here for Dendra2 in its green state (left) and in its red state (right). The PSF and the beam profiles of the various lasers used in the simulated experiment are shown on the left column.
