# Supplementary material for: In cellulo Evaluation of Phototransformation Quantum Yields in Fluorescent Proteins Used As Markers for Single-Molecule Localization Microscopy
Source: PLoS One. 2014 Jun 10;9(6):e98362. doi: 10.1371/journal.pone.0098362 (PMC4051587; doi:10.1371/journal.pone.0098362)
Supplement: Figure S2 — Representative frames from a PALM dataset of a “blank” PVA sample devoid of Dendra2 molecules (left) and a PVA sample containing Dendra2 (right). (PDF) [file pone.0098362.s002.pdf]

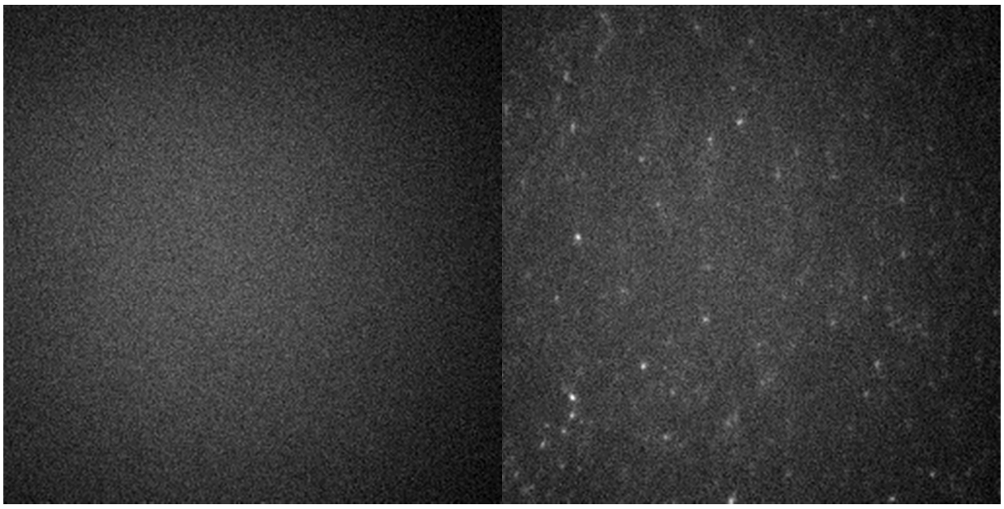

Figure S2: Representative frames from a PALM dataset of a “blank” PVA sample devoid of Dendra2 molecules (left) and a PVA sample containing Dendra2 (right). No blinking molecule can be noticed on the left image. Images are acquired under the same experimental conditions.
