# Supplementary material for: In cellulo Evaluation of Phototransformation Quantum Yields in Fluorescent Proteins Used As Markers for Single-Molecule Localization Microscopy
Source: PLoS One. 2014 Jun 10;9(6):e98362. doi: 10.1371/journal.pone.0098362 (PMC4051587; doi:10.1371/journal.pone.0098362)
Supplement: Figure S3 — Representative example of spot's clustering by the k-means algorithm. (PDF) [file pone.0098362.s003.pdf]

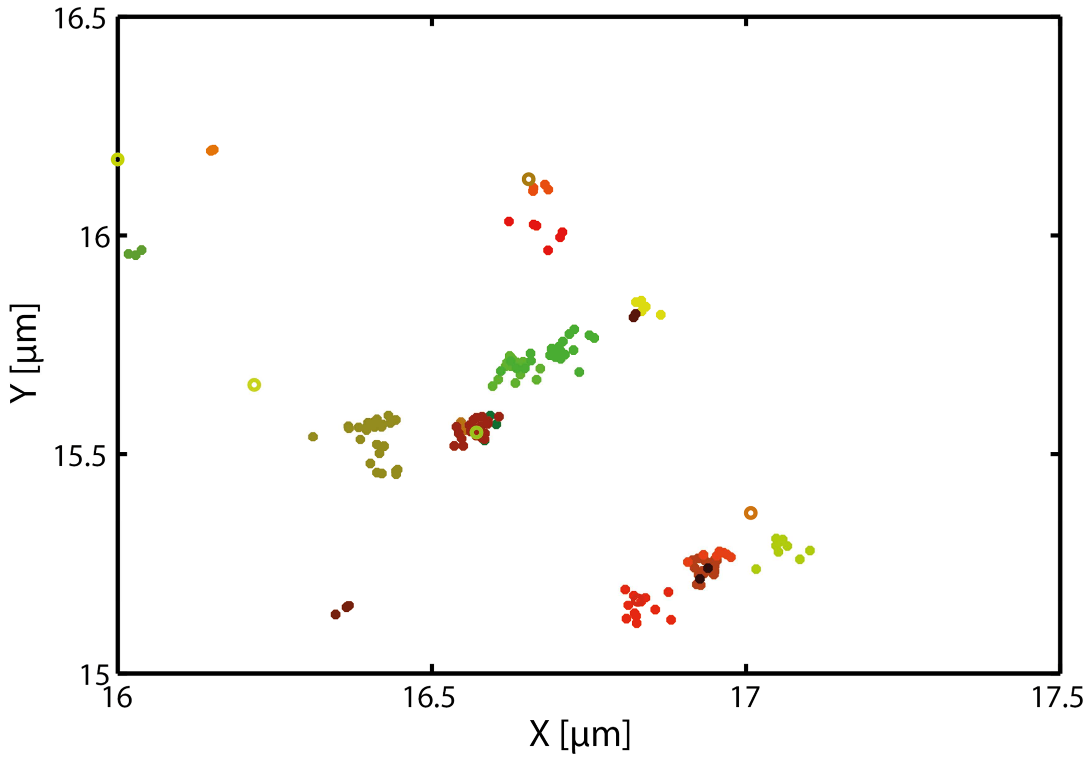

Figure S3: Representative example of spots clustering by the *k-means* algorithm. Groups of localized spots assigned to a single molecule are differently colored. Spatially overlapping groups correspond to single molecules separated in time (> 4 s). Spots with annular shape correspond to single molecules that appeared only once.
