# Supplementary material for: In cellulo Evaluation of Phototransformation Quantum Yields in Fluorescent Proteins Used As Markers for Single-Molecule Localization Microscopy
Source: PLoS One. 2014 Jun 10;9(6):e98362. doi: 10.1371/journal.pone.0098362 (PMC4051587; doi:10.1371/journal.pone.0098362)
Supplement: Figure S4 — Absorption spectrum of Dendra2 in its green state collected at acidic pH, after deconvolution of a residual contribution by the chromophore in its anionic state. (PDF) [file pone.0098362.s004.pdf]

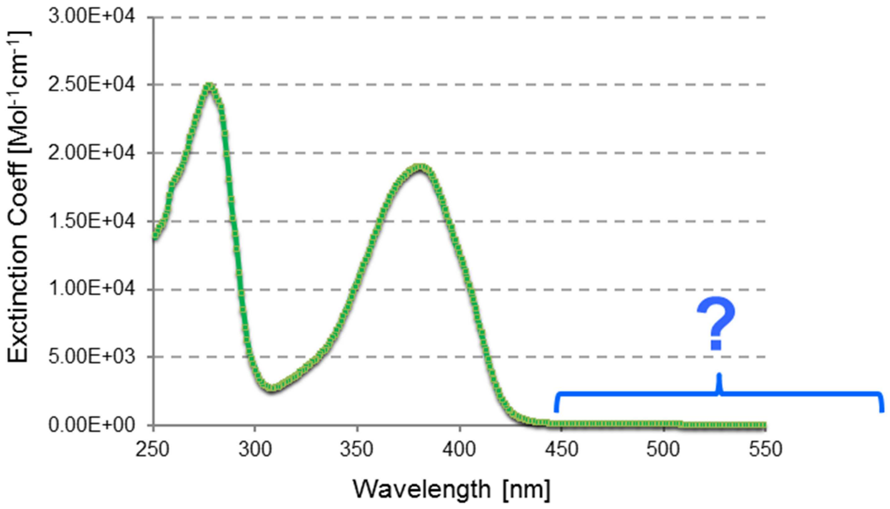

Figure S4: Absorption spectrum of Dendra2 in its green state collected at acidic pH, after deconvolution of a residual contribution by the chromophore in its anionic state. This spectrum is representative of the Dendra2 chromophore in its green neutral state, and is taken as the action spectrum for photoconversion. Extinction coefficients in the wavelength range corresponding to the used readout laser beam are close to zero and cannot be precisely determined.
