# Supplementary material for: In cellulo Evaluation of Phototransformation Quantum Yields in Fluorescent Proteins Used As Markers for Single-Molecule Localization Microscopy
Source: PLoS One. 2014 Jun 10;9(6):e98362. doi: 10.1371/journal.pone.0098362 (PMC4051587; doi:10.1371/journal.pone.0098362)
Supplement: Figure S5 — Rendered PALM image of the simulated microtubule sample (top) and 2D profile of the simulated laser beam (bottom). (PDF) [file pone.0098362.s005.pdf]

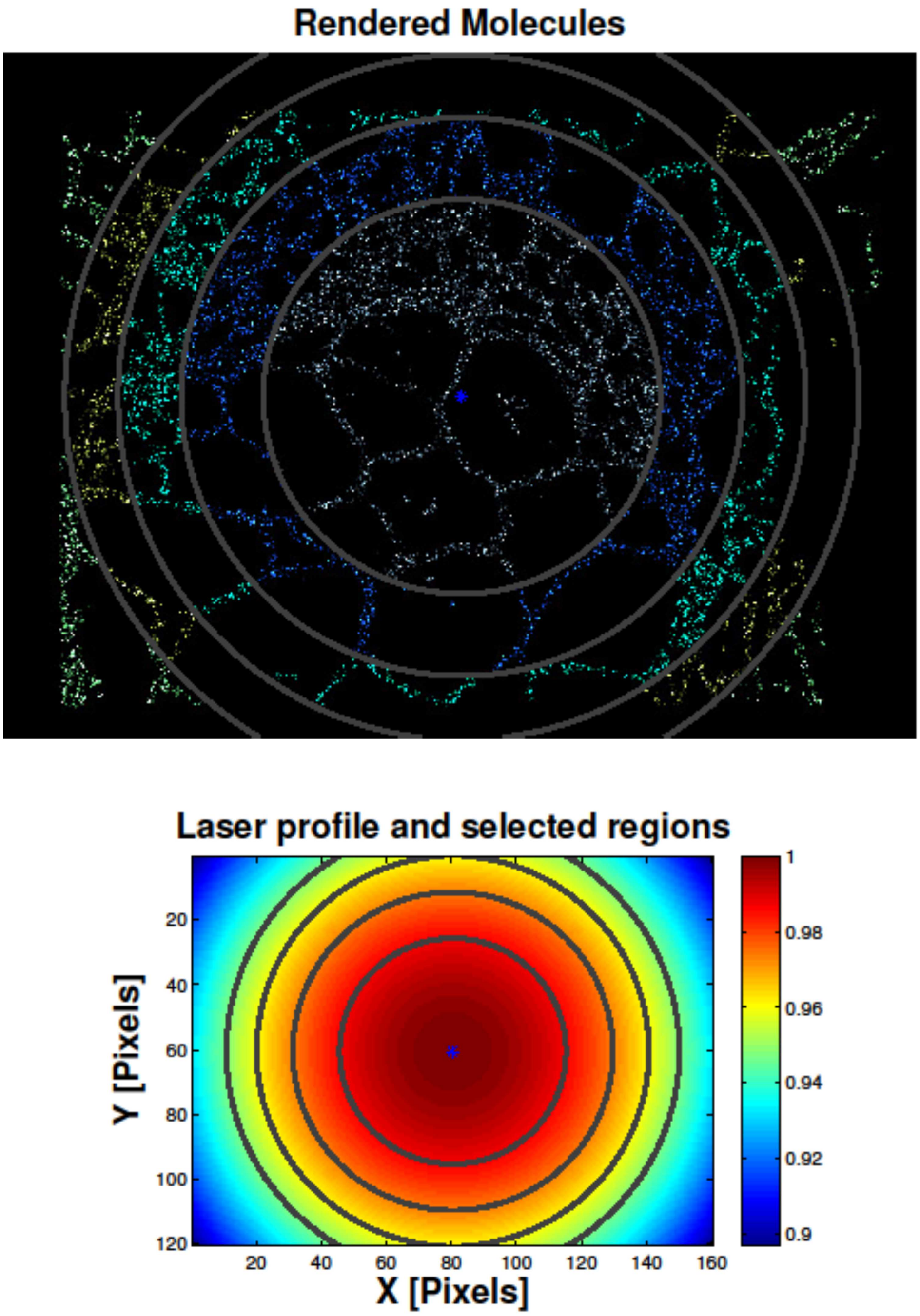

Figure S5: Rendered PALM image of the simulated microtubule sample (top) and 2D profile of the simulated laser beam (bottom). Molecules are sorted in five concentric regions (shown in different colors) in which the laser power density can be considered homogeneous.
