# Supplementary material for: In cellulo Evaluation of Phototransformation Quantum Yields in Fluorescent Proteins Used As Markers for Single-Molecule Localization Microscopy
Source: PLoS One. 2014 Jun 10;9(6):e98362. doi: 10.1371/journal.pone.0098362 (PMC4051587; doi:10.1371/journal.pone.0098362)
Supplement: Figure S6 — Final τc -curve extracted from a PALM data set generated with the parameters shown in Table S1. (PDF) [file pone.0098362.s006.pdf]

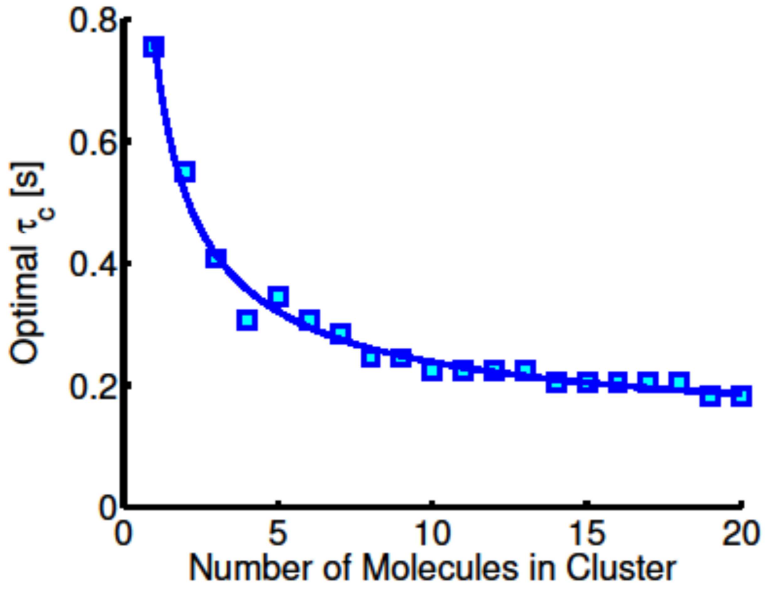

Figure S6: Final τ_c-curve extracted from a PALM data set generated with the parameters shown in Table S1.
