# Supplementary material for: In cellulo Evaluation of Phototransformation Quantum Yields in Fluorescent Proteins Used As Markers for Single-Molecule Localization Microscopy
Source: PLoS One. 2014 Jun 10;9(6):e98362. doi: 10.1371/journal.pone.0098362 (PMC4051587; doi:10.1371/journal.pone.0098362)
Supplement: Figure S7 — (A) Representative example of single-molecule trace reconstruction. (B) correlation histogram between the retrieved single-molecule traces and true simulated traces. (C) correlation histogram between the retrieved single-molecule traces and rounded true simulated traces. (PDF) [file pone.0098362.s007.pdf]

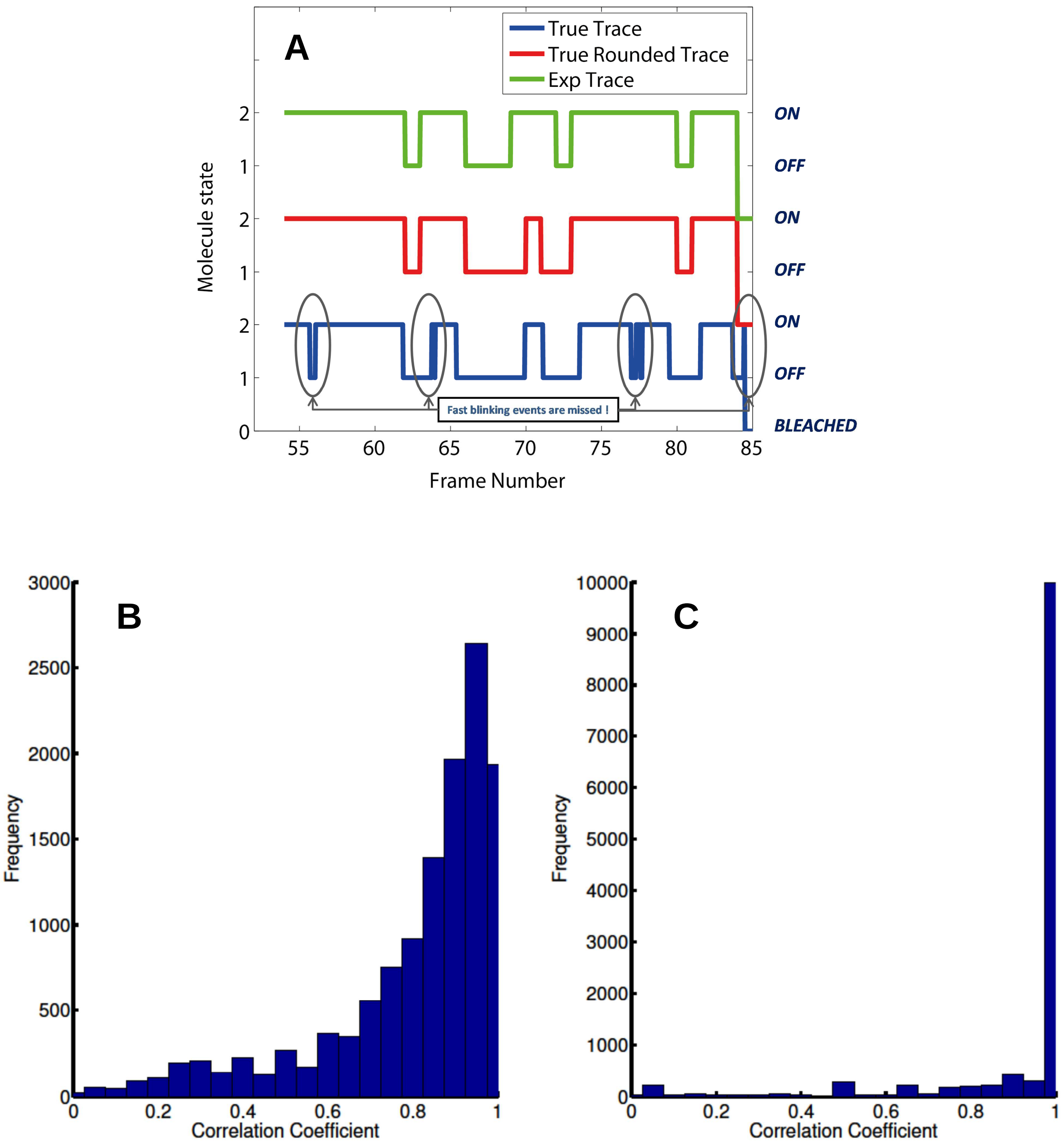

Figure S7: (A) Representative example of single-molecule trace reconstruction: bottom: true trace, middle: rounded true trace, top: retrieved experimental trace. It clearly appears that fast blinking events cannot be properly recovered (B) correlation histogram between the retrieved single molecule traces and true simulated traces. (C) correlation histogram between the

retrieved single molecule traces and rounded true simulated traces. Comparison between the two histograms highlight the loss of accuracy in single-molecule trace recovery due to the limited time-resolution of PALM experiments
