# Supplementary material for: In cellulo Evaluation of Phototransformation Quantum Yields in Fluorescent Proteins Used As Markers for Single-Molecule Localization Microscopy
Source: PLoS One. 2014 Jun 10;9(6):e98362. doi: 10.1371/journal.pone.0098362 (PMC4051587; doi:10.1371/journal.pone.0098362)
Supplement: Figure S8 — Effects of limited time-resolution on the determination of photo transformation yields. (PDF) [file pone.0098362.s008.pdf]

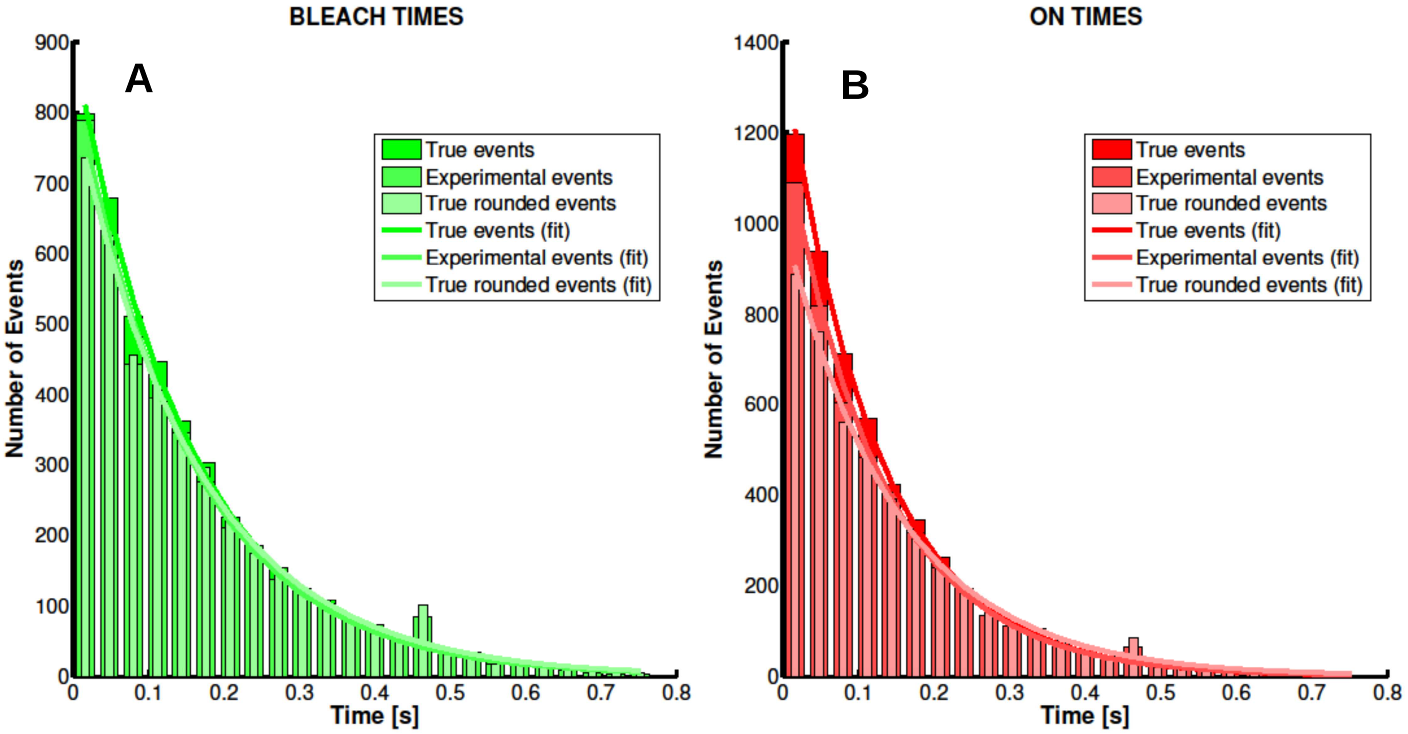

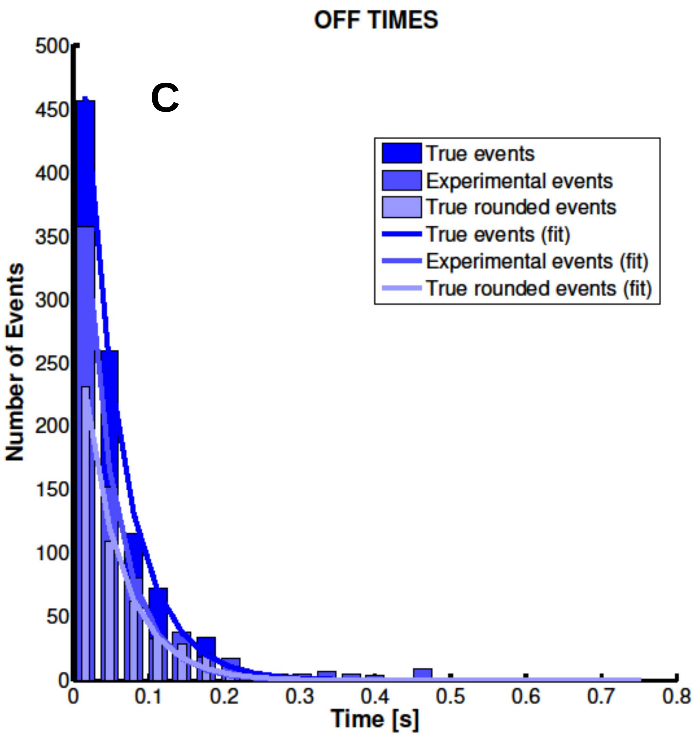

Figure S8: Effects of limited time-resolution on the determination of photo transformation yields. Histograms derived from true traces, rounded true traces, and retrieved experimental traces are shown as well as associated fitted decays. (**A**): N_{bleach}; (**B**): N_{on} (**C**): N_{off} . Data were generated with parameters of Table S1. Rounding of the true traces produce histograms close to those from experimental traces. The more severe distortion of the experimental fitted decay relative to the true trace decay in the case of the N_{on} histograms is apparent.
