# Supplementary material for: In cellulo Evaluation of Phototransformation Quantum Yields in Fluorescent Proteins Used As Markers for Single-Molecule Localization Microscopy
Source: PLoS One. 2014 Jun 10;9(6):e98362. doi: 10.1371/journal.pone.0098362 (PMC4051587; doi:10.1371/journal.pone.0098362)
Supplement: Figure S9 — Representative Nbleach histograms for tumbling molecules (A), fixed- molecules under circularly polarized laser (B), and fixed molecules under linearly polarized laser (C). (PDF) [file pone.0098362.s009.pdf]

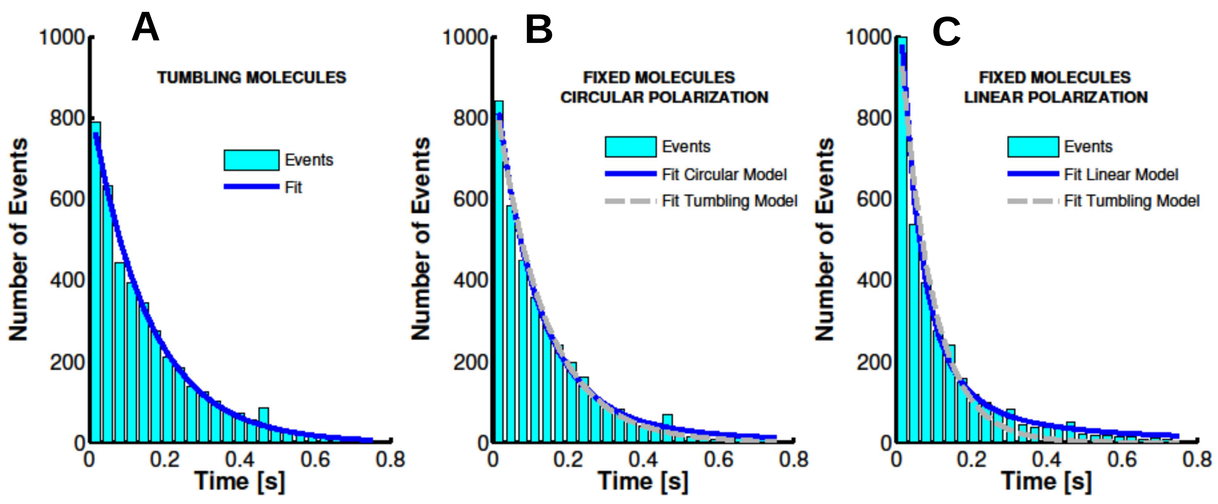

Figure S9: Representative N_{bleach} histograms for tumbling molecules (**A**), fixed-molecules under circularly polarized laser (**B**), and fixed molecules under linearly polarized laser (**C**). Data were generated with parameters of Table S1. The increase concavity of the histograms in going from tumbling molecules to fixed molecules under a linearly polarized laser is apparent. Fitting histograms from fixed molecules using the tumbling model (grey dashed lines) produces lower quality results.
