# Supplementary material for: In cellulo Evaluation of Phototransformation Quantum Yields in Fluorescent Proteins Used As Markers for Single-Molecule Localization Microscopy
Source: PLoS One. 2014 Jun 10;9(6):e98362. doi: 10.1371/journal.pone.0098362 (PMC4051587; doi:10.1371/journal.pone.0098362)
Supplement: Figure S10 — Plots of cumulative activation for tumbling molecules (A), fixed molecules under circularly polarized laser (B), and fixed molecules under linearly polarized laser (C). (PDF) [file pone.0098362.s010.pdf]

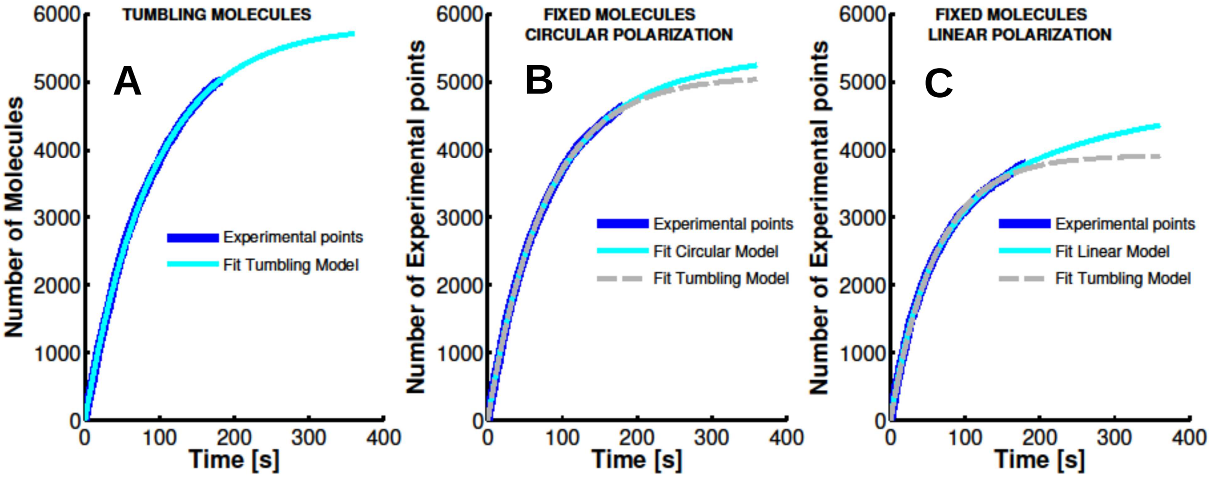

Figure S10: Plots of cumulative activation for tumbling molecules (**A**), fixed molecules under circularly polarized laser (**B**), and fixed molecules under linearly polarized laser (**C**). Data were generated with parameters of Table S1. Fitting activation plots from fixed molecules using the tumbling model (grey dashed lines) produces lower quality results.
