# Supplementary material for: In cellulo Evaluation of Phototransformation Quantum Yields in Fluorescent Proteins Used As Markers for Single-Molecule Localization Microscopy
Source: PLoS One. 2014 Jun 10;9(6):e98362. doi: 10.1371/journal.pone.0098362 (PMC4051587; doi:10.1371/journal.pone.0098362)
Supplement: Figure S11 — Errors in retrieved photobleaching yield, on-off blinking rate and photoconversion yield (or brightness) upon varying the input off-on blinking yield. (PDF) [file pone.0098362.s011.pdf]

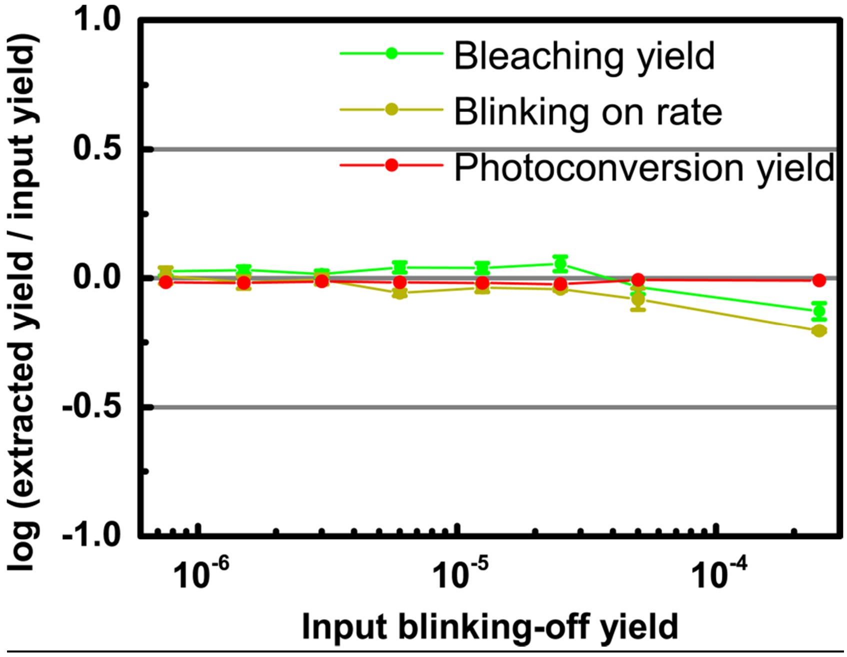

Figure S11: Errors in retrieved photobleaching yield, *on-off* blinking rate and photoconversion yield (or brightness) upon varying the input *off-on* blinking yield.
