# Supplementary material for: In cellulo Evaluation of Phototransformation Quantum Yields in Fluorescent Proteins Used As Markers for Single-Molecule Localization Microscopy
Source: PLoS One. 2014 Jun 10;9(6):e98362. doi: 10.1371/journal.pone.0098362 (PMC4051587; doi:10.1371/journal.pone.0098362)
Supplement: Figure S12 — Rendered PALM image of Dendra2-β-actin in a fixed HeLa cell under widefield illumination conditions. (PDF) [file pone.0098362.s012.pdf]

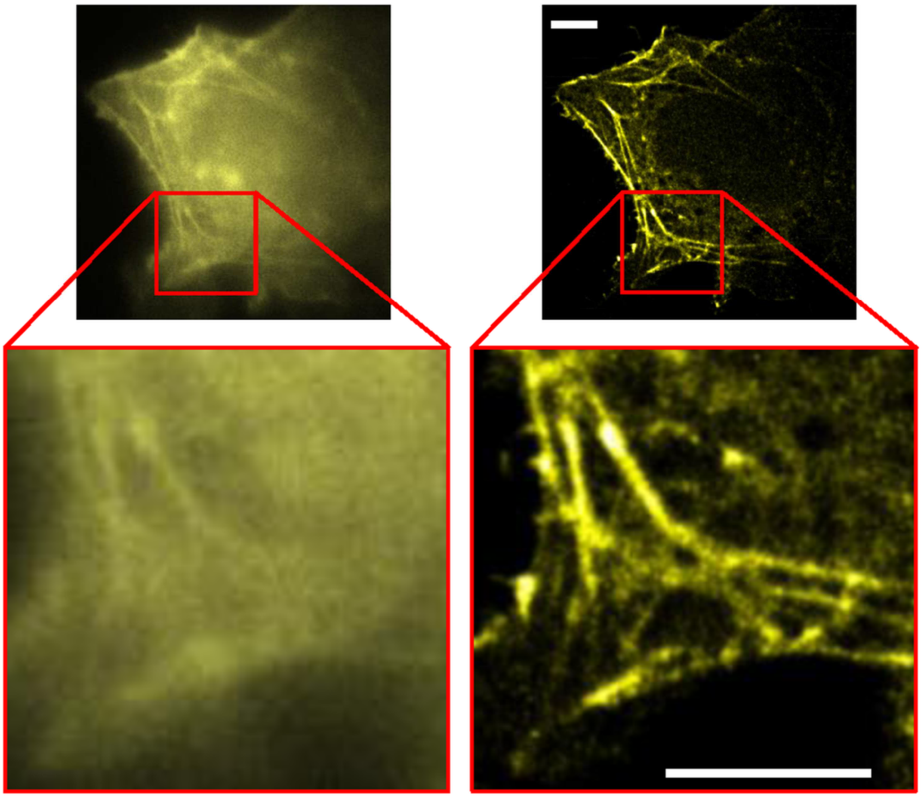

Figure S12: Rendered PALM image of Dendra2-β-actin in a fixed HeLa cell under widefield illumination conditions (Left: diffraction limited image; Right: PALM image. Scale bar, 5 μm.
