# Supplementary material for: In cellulo Evaluation of Phototransformation Quantum Yields in Fluorescent Proteins Used As Markers for Single-Molecule Localization Microscopy
Source: PLoS One. 2014 Jun 10;9(6):e98362. doi: 10.1371/journal.pone.0098362 (PMC4051587; doi:10.1371/journal.pone.0098362)
Supplement: Appendix S1 — Excitation rate of fixed molecules. (PDF) [file pone.0098362.s013.pdf]

Appendix S1: Excitation rate of fixed molecules

Let's consider a fluorophore of fixed orientation defined by spherical angles θ and φ. We ask what is the rate of excitation of that molecule by a circularly or linearly polarized laser. Let's assume that the excitation rate of a rapidly tumbling molecule is *k*.

We start with a linearly polarized laser, of coordinates $\begin{pmatrix}1\\0\\0\end{pmatrix}$. The fluorophore dipole has coordinates $\begin{pmatrix}\cos\theta\cos\varphi\\ \cos\theta\sin\varphi\\ \sin\theta\end{pmatrix}$. The sinus of the angle ϕ between the laser and the fluorophore dipole is given by the vectorial product.

$$\sin^2\phi = \left\| \begin{pmatrix}\cos\theta\cos\varphi\\ \cos\theta\sin\varphi\\ \sin\theta\end{pmatrix} \wedge \begin{pmatrix}1\\0\\0\end{pmatrix} \right\|^2 = \sin^2\theta + \cos^2\theta\sin^2\varphi$$

Therefore: $\cos^2\phi = 1 - \sin^2\theta - \cos^2\theta\sin^2\varphi = \cos^2\theta\cos^2\varphi$

The rate of excitation of the chromophore is given by:

$$k_{\theta,\varphi} = k'\cos^2\phi = k'\cos^2\theta\cos^2\varphi$$

knowing that $\langle k_{\theta,\varphi}\rangle = k$ (because the average over a population of randomly oriented fixed molecules is equivalent to a single tumbling molecule). Now:

$$\langle k_{\theta,\varphi}\rangle = \int k_{\theta,\varphi}\, p(\theta,\varphi)\, d\theta d\varphi \text{ with } \int p(\theta,\varphi)\, d\theta d\varphi = 1$$

So now comes the question of what is $p(\theta,\varphi)$ for a random distribution of molecules over half a sphere:

$$\int p(\theta,\varphi)\, d\theta d\varphi = 1 = \alpha\int \cos(\theta)\, d\theta d\varphi = \alpha\int_0^{\pi/2}\cos(\theta)\, d\theta \int_0^{2\pi} d\varphi = 2\pi\alpha$$

Hence: $\alpha = 1/2\pi$ and $p(\theta,\varphi) = 1/2\pi \times \cos(\theta)$

Therefore, in order to calculate *k'* we have:

$$\langle k_{\theta,\varphi} \rangle = k = 1/2\pi \times k' \int \cos^2(\theta)\cos^2(\varphi)\cos(\theta)d\theta d\varphi$$

$$k = 1/2\pi \times k' \int \cos^2(\theta)\cos(\theta)d\theta \int \cos^2(\varphi)d\varphi$$

The first integral is equal to:

$$\int (1-\sin^2(\theta))\cos(\theta)d\theta = \left[\int_0^{\pi/2}\cos(\theta)d\theta - \int_0^{\pi/2}\sin^2(\theta)\cos(\theta)d\theta\right] = \left[1-\frac{1}{3}[\sin^3(\theta)]_0^{\pi/2}\right] = 2/3$$

The second integral is equal to:

$$\int_0^{2\pi}\cos^2(\varphi)d\varphi = \int_0^{2\pi}\sin^2(\varphi)d\varphi = 1/2\times\left(\int_0^{2\pi}\cos^2(\varphi)d\varphi + \int_0^{2\pi}\sin^2(\varphi)d\varphi\right) = 1/2\times 2\pi = \pi$$

Overall: $k = 1/2\pi \times k' \times 2/3 \times \pi = k'/3$

Thus in the case of a linearly polarized laser:

$$k_{\theta,\varphi} = 3k\cos^2\theta\cos^2\varphi$$

In the case of a circularly polarized laser, we have to average over the angle φ, and

$$k_{\theta,\varphi} = 3k\cos^2\theta\langle\cos^2\varphi\rangle = 3/2\times k\cos^2\theta$$

$$k_{\theta,\varphi} = 3/2\times k\cos^2\theta$$
