# Supplementary material for: In cellulo Evaluation of Phototransformation Quantum Yields in Fluorescent Proteins Used As Markers for Single-Molecule Localization Microscopy
Source: PLoS One. 2014 Jun 10;9(6):e98362. doi: 10.1371/journal.pone.0098362 (PMC4051587; doi:10.1371/journal.pone.0098362)
Supplement: Discussion S1 — Underestimation of blinking-off quantum yield. (PDF) [file pone.0098362.s014.pdf]

Discussion S1

Underestimation of blinking-off quantum yield

The sub-optimal determination of the blinking-off yield is intriguing and deserves careful analysis. The first issue is that the N_{on} histogram is governed by the sum of the blinking-off and bleaching yields, implying that the blinking-off yield is retrieved by subtracting the bleaching yield and therefore incorporates the error made in determining the latter through analysis of the N_{bleach} histogram. The second issue is more prominent and is due to the limited time-resolution of typical PALM data acquisition. In our simulation, the average molecular lifetime before either reversible or irreversible fluorescence loss amounts to 4.8 frames whereas that before on-state recovery is 2.4 frames. As a consequence, substantial rounding errors in estimating molecular lifetimes are made. It is interesting to compare the retrieved N_{on} , N_{off} and N_{bleach} histograms with the histograms that can be built from the knowledge of the “true” molecular fluorescence traces (Figure S7A). Inspection of this figure shows that for all 3 histograms a significantly reduced number of molecules in most bins are observed, as compared to the “true” histograms. However, only the determination of the blinking-off quantum yield appears to be severely impacted. This behavior can be explained in the following way: the lower total number of retrieved events for all three histograms results from the fact that short events are not detected. In the case of bleaching and *off-on* blinking, missed events do not result in substantial distortions of the N_{bleach} and N_{off} histograms. The situation is different in the case of *off-on* blinking because in this case short on-times tend to be detected as long on-times: on-times shorter than the frame-time get artificially lengthened to one frame-time if the following off-time is longer than the frame-time, or get merged with the next on-time if the following off-time is shorter than the frame-time. This behavior is exacerbated when the sensitivity of the detection is high. On the

contrary, short off-times are typically ignored rather than artificially lengthened, unless the following on-time is so short that it remains undetected due to insufficient photon output. The validity of this interpretation is confirmed by comparing histograms of the true molecular traces with those obtained by simply “rounding” the latter to simulate the effect of the limited time-resolution: histograms of the rounded true traces resemble, and in fact exacerbate the trends observed in the retrieved experimental traces. Their fitting consistently results in a large underestimation of the blinking-off quantum yield, but in a small deviation of the bleaching yield and *off-on* blinking rate (Table S2).

Another point is worth noticing: in the presence of very low blinking-on rates (like e.g. observed with organic dyes in dSTORM) and a high density of labeling, application of τ_c splitting could be expected to strongly bias N_{off} histograms towards short off-times. Further simulations will be needed to estimate the magnitude of this effect.
