# Supplementary material for: In cellulo Evaluation of Phototransformation Quantum Yields in Fluorescent Proteins Used As Markers for Single-Molecule Localization Microscopy
Source: PLoS One. 2014 Jun 10;9(6):e98362. doi: 10.1371/journal.pone.0098362 (PMC4051587; doi:10.1371/journal.pone.0098362)
Supplement: Scripts S1 — Matlab scripts for simulation of PALM data sets and for the extraction of phototransformation yields. (ZIP) [file pone.0098362.s015.zip › Help.rtf]

	In cellulo Evaluation of Phototransformation Quantum Yields in Fluorescent Proteins Used as Markers for Single-Molecule Localization Microscopy.

Sergiy Avilov, Romain Berardozzi, Mudalige S. Gunewardene, Virgile Adam, Samuel T. Hess and Dominique Bourgeois.

Description of Matlab scripts.
The provided scripts should first be unzipped.
The scripts are compatible with Matlab version R2013b.
The scripts are not guaranteed for absence of bugs !
For further help please contact dominique.bourgeois@ibs.fr
The scripts can be divided into three parts:
A: simulation of PALM data
ð	Run the script: simulate_palm_vsn9_main.m in the main directory
ð	Scripts for this part are found in the folder named PALM_SIMULATIONS
ð	Other examples of simulation scripts are found in PALM_SIMULATIONS\OTHER_SIMULATIONS 
B: localization of PALM data 
ð	Run the script: localize_palm_data_vsn8.m in the main directory (localization)
ð	Run the script: apply_tolerances_vsn2.m in the main directory (tolerances)
ð	Scripts for this part are found in the folder named PALM_LOCALIZATION
C: extraction of photo transformation yields
ð	Run the script: extract_yields_exp_data_vsn10_main.m in the main directory 
ð	Scripts for this part are found in the folder named YIELD_EXTRACTION
ð	The script extract_yields_true_data_vsn10_main.m can be used to extract phototransformation yields directly from simulated data, without going through the localization step.
ð	The script Time_Trace_Examinator.m can be used to examine how blinking has been extracted from experimental data.
ð	The script Time_Trace_Correlator_Struc2.m does the same for simulation data that have been processed the whole way.
