# Supplementary figures and images for: In cellulo Evaluation of Phototransformation Quantum Yields in Fluorescent Proteins Used As Markers for Single-Molecule Localization Microscopy
Source: PLoS One. 2014 Jun 10;9(6):e98362. doi: 10.1371/journal.pone.0098362 (PMC4051587; doi:10.1371/journal.pone.0098362)

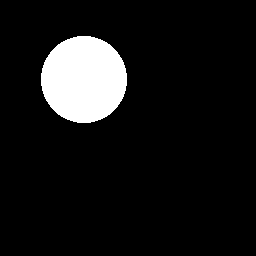

Supplement: Scripts S1 — Matlab scripts for simulation of PALM data sets and for the extraction of phototransformation yields. (ZIP) [file pone.0098362.s015.zip › PALM_SIMULATIONS/PATTERNS/Disk1.tif]

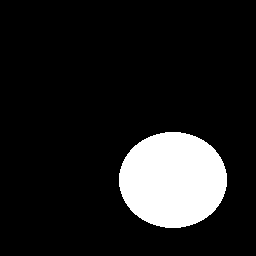

Supplement: Scripts S1 — Matlab scripts for simulation of PALM data sets and for the extraction of phototransformation yields. (ZIP) [file pone.0098362.s015.zip › PALM_SIMULATIONS/PATTERNS/Disk2.tif]

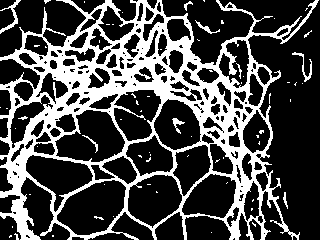

Supplement: Scripts S1 — Matlab scripts for simulation of PALM data sets and for the extraction of phototransformation yields. (ZIP) [file pone.0098362.s015.zip › PALM_SIMULATIONS/PATTERNS/Pattern_Microtubules.tif]

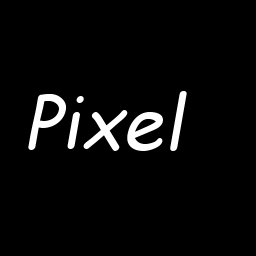

Supplement: Scripts S1 — Matlab scripts for simulation of PALM data sets and for the extraction of phototransformation yields. (ZIP) [file pone.0098362.s015.zip › PALM_SIMULATIONS/PATTERNS/Pattern_Pixel_4.tif]
