# Supplementary material for: In cellulo Evaluation of Phototransformation Quantum Yields in Fluorescent Proteins Used As Markers for Single-Molecule Localization Microscopy
Source: PLoS One. 2014 Jun 10;9(6):e98362. doi: 10.1371/journal.pone.0098362 (PMC4051587; doi:10.1371/journal.pone.0098362)
Supplement: Table S1 — Parameters used to generate simulated PALM data sets. (PDF) [file pone.0098362.s016.pdf]

Table S1: Parameters used to generate the PALM data set described at the beginning of the Results section of this paper.

Readout laser power	30 mW
Readout laser full width at half maximum	50 µm
Readout laser wavelength	561 nm
Laser polarization	Circular
Objective numerical aperture	1.49
Immersion oil index	1.51
Detection efficiency	4%
Detector Gain	30 counts/photon
Pixel Size	100×100 nm
Dark noise	2 photons/pixel/frame
Auto fluorescence	10 photons/pixel/frame
Frametime	30 ms
Number of frames	6000
Molecular density per μm^2	545
Fluorescence quantum yield	0.55
Photoconversion brightness	$4.28\ 10^{-4}$
Bleaching yield	5.10^{-6}
Blinking off yield	2.10^{-5}
Blinking on rate	$20\ s^{-1}$
Mean signal to noise#	60.8
Mean photon output#	755 photons/molecule
Mean localization precision#	7 nm

Resulting output parameters.
