# Supplementary material for: In cellulo Evaluation of Phototransformation Quantum Yields in Fluorescent Proteins Used As Markers for Single-Molecule Localization Microscopy
Source: PLoS One. 2014 Jun 10;9(6):e98362. doi: 10.1371/journal.pone.0098362 (PMC4051587; doi:10.1371/journal.pone.0098362)
Supplement: Table S2 — Comparison between phototransformation yields retrieved from true single-molecule traces, rounded true traces and experimental traces. (PDF) [file pone.0098362.s017.pdf]

Table S2: Comparison between phototransformation yields retrieved from true single-molecule (SM) traces, rounded true traces and experimental traces.

	**True**	**True SM traces#**	**Rounded true SM traces&**	**Experimental traces$**
ϕ_{bleach}	2×10^{-5}	$(1.98\pm0.02)\times10^{-5}$	$(1.82\pm0.03)\times10^{-5}$	$(2.01\pm0.05)\times10^{-5}$
$\phi_{on\text{-}off}$	5×10^{-6}	$(5.19\pm0.32)\times10^{-6}$	$(2.14\pm0.42)\times10^{-6}$	$(3.61\pm0.07)\times10^{-6}$
$k_{off\text{-}on}$ [s^{-1}]	20	20.28±0.34	18.4±0.60	21.9±0.66
φ_A	4.28×10^{-4}	$(4.21\pm0.007)\times10^{-4}$	$(4.21\pm0.007)\times10^{-4}$	$(4.10\pm0.004)\times10^{-4}$

Given standard deviations correspond to histogram fitting errors.

True SM traces refer to the single molecule traces output by our PALM simulation software. In these traces, molecules are either on, off or bleached and the time resolution is 0.1 µs.

& Rounded true SM traces were obtained by assigning a constant state value to the true SM traces during each simulated frame (30 ms duration). The single molecule was assumed to be *on* during the entire frame if it was *on* for at least 20% of the time during that frame. Otherwise, it was assumed to be off during the entire frame or bleached at the beginning of the frame.

Experimental traces refer to traces recovered upon applying the processing pipeline described in Figure 2.
