# Supplementary material for: In cellulo Evaluation of Phototransformation Quantum Yields in Fluorescent Proteins Used As Markers for Single-Molecule Localization Microscopy
Source: PLoS One. 2014 Jun 10;9(6):e98362. doi: 10.1371/journal.pone.0098362 (PMC4051587; doi:10.1371/journal.pone.0098362)
Supplement: Table S3 — Comparison between phototransformation yields retrieved from simulated data sets with tumbling molecules and fixed molecules. (PDF) [file pone.0098362.s018.pdf]

Table S3: Comparison between phototransformation yields retrieved from simulated data sets with tumbling molecules and fixed molecules (circularly polarized laser and linearly polarized laser).

	True	Tumbling	Fixed (Circular)		Fixed (Linear)	
			Circular model	*Tumbling model*	*Linear model*	*Tumbling model*
ϕ_{bleach}	2×10^{-5}	$(2.01\pm0.05)\times10^{-5}$	$(2.17\times\pm0.06)10^{-5}$	$(2.31\pm0.05)\times10^{-5}$	$(2.92\pm0.11)\times10^{-5}$	$(3.65\pm0.19)\times10^{-5}$
$\phi_{on\text{-}off}$	5×10^{-6}	$(3.61\pm0.07)\times10^{-6}$	$(3.13\times\pm0.20)10^{-6}$	$(3.36\pm0.17)\times10^{-6}$ #	$(2.53\pm0.32)\times10^{-6}$	$(3.18\pm0.49)\times10^{-6}$ #
$k_{off\text{-}on}$ [s^{-1}]	20	21.9±0.66	23.9±0.79	23.9±0.83	26.2±1.20	26.4±1.10
Nmol	26272	24029±96	23308±140	19940±120	26098±130	16022±128
φ_A	4.28×10^{-4}	$(4.10\pm0.004)\times10^{-4}$	$(4.21\pm0.006)\times10^{-4}$	$(4.89\pm0.006)\times10^{-4}$	$(3.67\pm0.005)\times10^{-4}$	$(6.03\pm0.008)\times10^{-4}$

Given standard deviations correspond to histogram fitting errors.

When the model corresponding to tumbling molecules is applied to data sets generated with fixed molecules, $\phi_{on\text{-}off}$ yields values are retrieved that are closer to the true values than when the correct model is used due to compensating errors.
